# Targeting ferroptosis in breast cancer

**DOI:** 10.1186/s40364-020-00230-3

**Published:** 2020-11-05

**Authors:** Zhaoqing Li, Lini Chen, Cong Chen, Yulu Zhou, Dengdi Hu, Jingjing Yang, Yongxia Chen, Wenying Zhuo, Misha Mao, Xun Zhang, Ling Xu, Linbo Wang, Jichun Zhou

**Affiliations:** 1 Department of Surgical Oncology, Sir Run Run Shaw Hospital, Zhejiang University, Hangzhou, 310000 Zhejiang China; 2 Cancer Institute (Key Laboratory of Cancer Prevention and Intervention, China National Ministry of Education), 2nd Affiliated Hospital, School of Medicine, Zhejiang University, 310009 Hangzhou, Zhejiang China; 3 Biomedical Research Center and Key Laboratory of Biotherapy of Zhejiang Province, 310000 Hangzhou, Zhejiang China; 4 Cixi People’s Hospital Medical and Health Group, 315300 Ningbo, Zhejiang China

**Keywords:** Ferroptosis, Breast cancer, Regulated cell death, Crosstalk, Ferroptotic regulators, Historical perspective

## Abstract

Ferroptosis is a recently discovered distinct type of regulated cell death caused by the accumulation of lipid-based ROS. Metabolism and expression of specific genes affect the occurrence of ferroptosis, making it a promising therapeutic target to manage cancer. Here, we describe the current status of ferroptosis studies in breast cancer and trace the key regulators of ferroptosis back to previous studies. We also compare ferroptosis to common regulated cell death patterns and discuss the sensitivity to ferroptosis in different subtypes of breast cancer. We propose that viewing ferroptosis-related studies from a historical angle will accelerate the development of ferroptosis-based biomarkers and therapeutic strategies in breast cancer.

## Introduction

Breast cancer is the most common cancer among women, with 1.7 million people diagnosed worldwide and approximately half a million people deaths from this disease each year [1]. Although surgical resection, radiotherapy, chemotherapy, endocrine therapy and targeted therapy have been applied for treatment, the prognosis of patients with breast cancer is still not satisfactory [2]. Therefore, there is an urgent need to develop novel therapeutic management for these patients who require more precise intervention.

The term ferroptosis was coined in 2012 to describe an iron-dependent regulated form of cell death caused by the accumulation of lipid-based reactive oxygen species (ROS) [3, 4]. Morphologically, obvious shrinkage of mitochondria with an increased membrane density and reduction of mitochondrial cristae could be observed, distinguishing ferroptosis from other types of cell death, such as apoptosis, autophagy, and necrosis [5]. Ferroptosis is characterized by oxidation of polyunsaturated fatty acid-containing phospholipids, the presence of redox-active iron and loss of lipid peroxide repairing ability [3]. Numerous agents targeting corresponding molecules involved in ferroptosis have been developed, making it a promising therapeutic strategy for cancer. Although a definitive pathophysiological function of ferroptosis has yet to be clearly demonstrated, the roles of ferroptosis in human diseases have been established, such as neurodegeneration [6, 7], ischaemia reperfusion injury [8] and various kinds of cancer including breast cancer [9–12]. A wealth of studies have suggested that pharmacological modulation of this unique cell death modality, either by inhibiting or stimulating it, may yield significant clinical benefit for certain diseases.

Accumulating evidence indicates that ferroptotic cell death leads to tumour growth suppression. Targeting ferroptosis might be a promising anticancer strategy. Recent discoveries of ferroptosis-inducing agents and further identification of regulatory mechanisms and genes involved in ferroptosis serve as a foundation for developing strategies for targeting ferroptosis in cancer therapy. Therefore, a better understanding of the processes that regulate ferroptosis sensitivity should ultimately aid in the discovery of novel therapeutic strategies to improve cancer treatment.

Although ferroptosis was defined only a few years prior, traces of its existence have emerged in previous studies in the last several decades. In this review, we first briefly introduce the main characteristics of ferroptosis and compare it with the other four common types of regulated cell death. We then discuss the current status of ferroptosis-related studies in breast cancer and differences between different subtypes of breast cancer, along with an extensive historical study consistent with the current definition of ferroptosis in breast cancer. From a historical perspective, we discuss recent implications and applications of manipulations of the ferroptotic death pathway in breast cancer.

### What is ferroptosis?

**Fig. 1:**
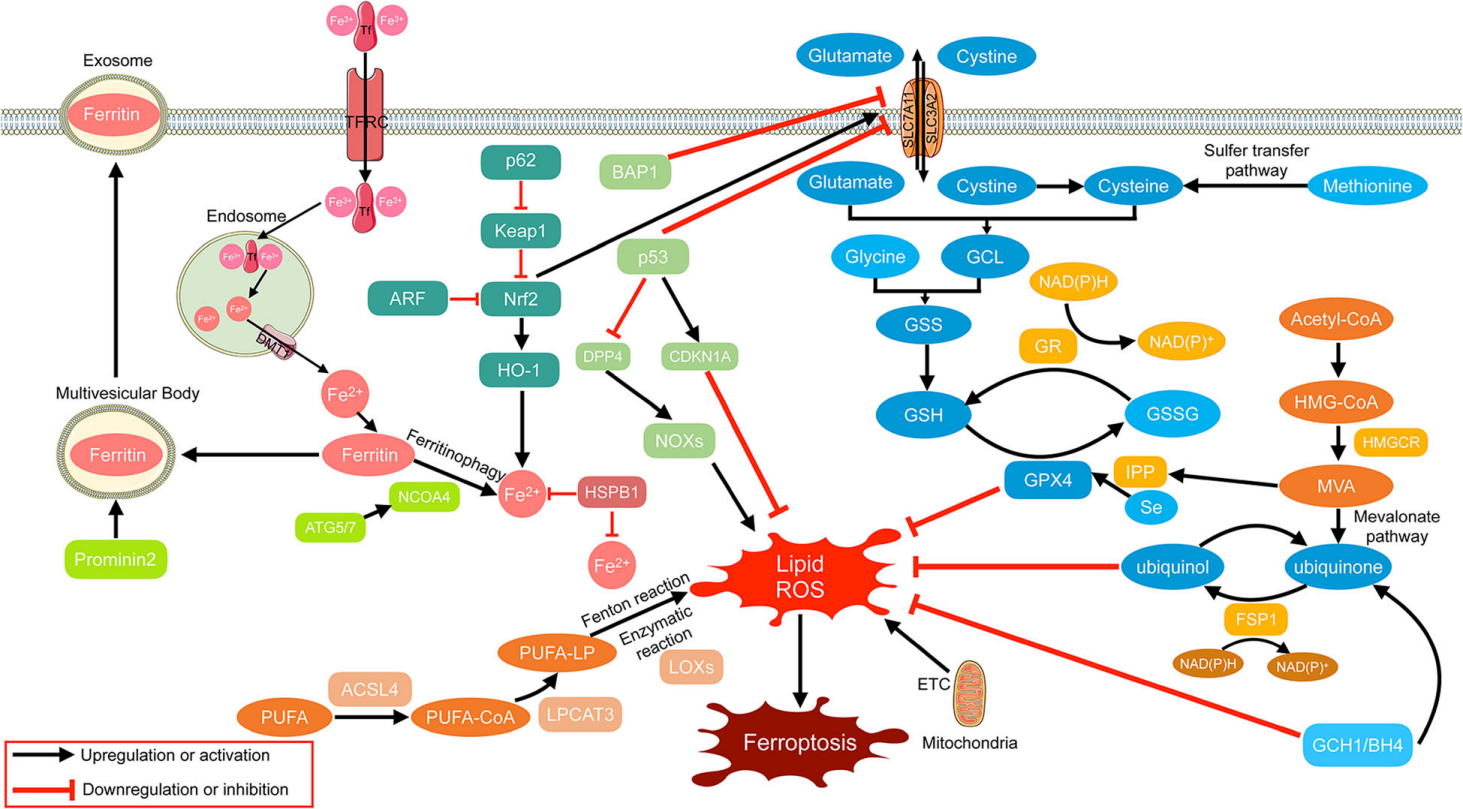
Overview of ferroptosis pathways. Transferrin (Tf) with two ferric iron (Fe^3+^) combines with TFRC and then enters the cell through endocytosis. In endosomes, ferric iron is reduced to ferrous iron (Fe^2+^) and released into the cytoplasm through DMT1. Fe^2+^ is stored as ferritin or function in an active loose state termed “labile iron pool”. Ferritin can be degraded via ferritinophagy mediated by NCOA4 to release Fe^2+^ into the cytoplasm. It can also be encapsulated into a multivesicular body mediated by Prominin2 and then transported out of the cell in the form of an exosome. The P62/Keap1/NRF2/HO-1 pathway, inhibited by ARF, contributes to the increase in Fe^2+^ by catabolizing heme. HSPB1 is a negative regulator of ferroptosis by reducing iron uptake and inhibiting ROS production. After being acetized by acyl-CoA synthetase (mainly ACSL4), PUFA-CoA is integrated into the cell membrane as PUFA-LP by LPCAT3 and then oxidized through the Fenton reaction mediated by Fe^2+^ and enzymatic reaction mediated mainly by LOXs into lipid ROS, which is the main killer in ferroptosis. ETC of the mitochondrial contributes to the generation of lipid ROS, while GPX4 and ubiquinol transform lipid peroxidation into nontoxic production. FSP1 is responsible for the conversion of ubiquinone into ubiquinol by consuming NADPH. HMGCR mediates the production of MVA from HMG-CoA derived from acetyl-CoA. MVA provides ubiquinone for FSP1 via the mevalonate pathway and IPP for combination of Se to GPX4. GPX4 exerts its role in a GSH-dependent manner. GSH is synthesized from glutamate, cysteine, and glycine. Cysteine is derived from methionine via the sulfur transfer pathway and cystine. The transporter system xc^−^, composed by SLC3A2 and SLC7A11, is the cystine/glutamate antiporter that imports cystine into cells while exporting glutamate. The subunit SLC7A11 is upregulated by NRF2 and downregulated by p53 and BAP1. P53 also suppress ferroptosis by downregulating DDP4 and upregulating CDKN1A

From 2001 to 2003, the Stockwell Lab performed a screen to identify compounds that kill ﻿cells ﻿engineered to be tumourigenic (harbouring the RAS mutant), without killing their isogenic parental precursors. One of the most efficient compounds was identified and named “erastin” after its ability to “Eradicate RAS-and Small T transformed cells” [13]. Subsequently, they identified RSL3, which was also named after its “oncogenic-RAS-selective lethal” property in 2008 [14]. In 2012, the term “ferroptosis” was coined to describe this iron-dependent, non-apoptotic form of cell death induced by erastin and RSL3 [4]. As ferroptosis became the focus of scientific research, an increasing number of mechanisms have been revealed. Three hallmarks of ferroptosis were described by Stockwell et al., i.e., ﻿the loss of lipid peroxide repair capacity by the phospholipid hydroperoxidase glutathione peroxidase-4 (GPX4), the availability of redox-active iron, and oxidation of polyunsaturated fatty acid (PUFA)-containing phospholipids [3], among which the latter is the main driver of ferroptotic death [15]. Thus, molecules that regulate the above processes may induce or suppress ferroptosis. For example, SLC7A11 (xCT), a subunit of system xc-, has been considered to be one of the most important regulators of ferroptosis by importing cysteine to synthesise GSH, which is the enzyme co-substrate of GPX4 in the conversion of lipid hydroperoxides to lipid alcohol [3]. NCOA4 induces ferroptosis by degrading ferritin and increasing cellular labile iron levels [16]. Another vital gene, Acyl-CoA Synthetase Long Chain Family Member (ACSL) 4, contributes to ferroptosis by enriching cellular membranes with long polyunsaturated n-6 fatty acids, which is subject to free radical or enzyme-mediated oxidation [17, 18]. The main pathways involved in ferroptosis are summarized and presented in Fig. 1 [3, 5, 19–21].

In addition to these molecular features, ferroptosis also exhibits morphological characteristics distinguishing it from other types of cell death. Ferroptosis does not induce chromatin agglutination or apoptotic bodies formation that occur in apoptosis, or plasma membrane breakdown that occurs in necroptosis, or formation of double-membraned autolysosomes that occurs in autophagy, or rupture of plasma membrane that occurs in pyroptosis; instead, it results in mitochondrial shrinkage and increased mitochondrial membrane density [4, 61]. Ferroptosis also has its unique biochemical features such as iron accumulation, lipid peroxidation, and elevated ΔΨm. Numerous small molecules inducing ferroptosis have been identified and divided into 5 classes. Class I FINs deplete GSH to inactivate GPX4; class II and III FINs inactivate GPX4 directly; class IV FINs induce iron overload; and others induce ferroptosis via other mechanisms. Ferroptosis inhibitors are divided as iron chelators, anti-oxidants, ROS formation inhibitors and others [5, 19, 22, 69, 71]. The main morphological features, regulating genes, inducers and inhibitors of ferroptosis, apoptosis, necroptosis, pyroptosis and autophagy-dependent cell death are listed in Table 1.

**Table 1:** The main morphological features, regulators, inducers and inhibitors of ferroptosis, apoptosis, necroptosis, autophagy-dependent cell death and pyroptosis

Cell Death	Defining Morphological Features	Biochemical Features	Key Regulators	Inducers	Inhibitors
Ferroptosis	Mitochondria become smaller, with increased mitochondrial membrane densities and reduced mitochondrial crista, outer mitochondrial membrane rupture and normal nucleus [4]	Iron accumulation; lipid peroxidation; ΔΨm elevated; caspase-independent [22]	GPX4 [23], p53 [24–26], HSPB1 [27, 28], SLC7A1 1[4], NRF2 [29–31], TFRC [14, 32], NCOA4 [16, 33], ACSL4 [17, 34–36], FSP1 [37]	GPX4 inactivation due to GSH depletion (class I FINs): erastin [4, 13], erastin derivatives (piperazine erastin [23], imidazole ketone erastin [15]), DPI2 [23], buthionine sulfoximine [23], sulfasalazine [4, 38], sorafenib [39], glutamate [4], cyst(e) inase [40], BAY 87–2243 [41, 42]; GPX4 inactivation/depletion (class II, III FINs): 1S,3R-RSL3 [4, 14, 23], DPI7/ML162, DPI10/ML210, DPI12, DPI13, DPI17, DPI18, DPI19 [23, 43], altretamine [44], FIN56 [45, 46], withaferin A [47], fluvastatin, lovastatin acid, simvastatin [48]; Iron loading (class IV FINs): hemoglobin [49], FeCl_2_ [49], hemin [47], (NH_4_)2Fe(SO_4_)_2_ [47], non-thermal plasma [50], salinomycin [51], amino acid depletion +Cornell dots [52], lapatinib + siramesine [12], FINO_2_ [45], BAY 11–7085 [53]; others: lanperisone [54], artemisinin derivatives [55, 56], CIL41, CIL56, CIL69, CIL70, CIL75, and CIL79 [46]	Iron chelators: desferoxamine [4], solamine [4], 2, 2- Bipyridyl [4]; Anti-oxidants: vitamin E [23], U0126 [4, 23], Trolox [4]; ROS formation inhibitors: ferrostatin-1 [4], SRS8–72 [4], SRS11–92, SRS12–45,SRS13–35, SRS13–37 [57], SRS16–86 [58]; Others: cycloheximide [4], aminooxyacetic acid [4], ebselen [4, 59], β-mercaptoethanol [4, 60]
Apoptosis	Plasma membrane blebbing; cellular and nuclear volume reduction; chromatin agglutination, nuclearfragmentation; formation of apoptotic bodies and cytoskeletal disintegration, no significant changes in mitochondrial structure [61]	Activation of caspases; DNA fragmentation; ΔΨm dissipation; phosphatidylserine exposure [22]	CASP2, CASP3, CASP6, CASP7, CASP8, CASP9, CASP10, CARD8, GZMB, HSPA1B, CARD6, NOX5, p53, Bax, Bak, BCL2 family (e.g., BAK1, BAX, BOK, BCL2L11, BBC3, PMAIP1, BID, BCL2, BCL2L1, MCL1, BCL2L2, and BCL2L10), Bcl-XL [19, 62]	Extrinsic apoptosis: FASL, DCC, Intrinsic apoptosis: multiple intracellular stress conditions (e.g. DNA damage, cytosolic Ca^2+^ overload) [61]	Inhibitors of apoptosis (IAPs): XIAP, c-IAP1, c-IAP2, ILP-2, ML-IAP/livin, NAIP, Bruce/Apollon, survivin [63]; caspase inhibitors: Z-VAD-FMK, emricasan, Q-VD-OPh, Z-VAD (OH)- FMK, Z-DEVD-FMK, Z-VDVAD-, ivachtin, Q-DEVD-OPh, Ac-DEVD-CHO, Z- IETD-FMK, Q-LEHD-OPh [22]
Necroptosis	Plasma membrane breakdown, generalized swelling of the cytoplasm and organelles, moderate chromatin condensation [61]	Drop in ATP levels; activation of RIPK1, RIPK3, and MLKL; cytosolic necrosome formation [5, 22]	RIP1, RIP3, MLKL, ESCRT-III, cIAPs, LUBAC, PPM1B, and AURKA [19, 22, 64]	TNFα, z-VAD-fmk [19]	RIP1 inhibitor: Necrostatin1 (Nec-1 )[65]; MLKL inhibitor: necrosulfonamide (NSA) [65]; RIPK3 inhibitors: GSK872, HS-1371 [65]
Autophagy-dependent cell death	Formation of double-membraned autolysosomes, including macroautophagy, microautophagy and chaperone-mediated autophagy [61]	MAP 1 LC3B-I to MAP 1LC3B-II conversion; increased autophagic flux and lysosomal activity [5, 22]	Beclin 1, ATG family proteins, LC3, DRAM3, TFEB, Na^+^/K^+^-ATPase, AMPK, mTOR [5, 19, 22, 66]	Rapamycin, lithium, sodium, valproate, carbamazepine [67]	Non-selective PI3K inhibitors: 3-methyladenine, LY294002, wortmannin; Selective VPS34 inhibitors: PIK-III, compound 31, SAR 405, Vps34-In1; Specific ULK1 inhibitors: MRT68921, MRT67307, SBI-0206965; Specific Beclin1 inhibitors: Spautin-1 Lysosome inhibitors: chloroquine, hydrochloroquin; lysosomal inhibitor: Chloroquine; H^+^-ATPase inhibitors: bafilomycin A1, concanamycin A; USP10 and USP13 inhibitor: spautin 1 [22, 68]
Pyroptosis	Lack of cell swelling; rupture of plasma membrane; bubbling; moderate chromatin condensation [61]	Activation of CASP1, CASP4, CASP5 (CASP1 and CASP11 in mice) and GSDMD; GSDMD cleavage; GSDMD-N–induced pore formation; IL1β and IL18 release [69]	CASP1, CASP4, CASP5 (CASP1 and CASP11 in mice) and GSDMD, GPX4, ESCRT-III, PKA [70]	Metformin, anthocyanin, DHA, DPP8/9 inhibitor, α-NETA, cisplatin, paclitaxel, iron, L61H10, BI2536, lobaplatin, doxorubicin [69]	CASP1 inhibitors: Ac-YVAD-cmk, Z-YVAD (OMe)-FMK, VX765; CASP11 inhibitor: wedelolactone; NLRP3-inflammasome inhibitors: MCC950, isoliquiritigenin, glybenclamide, CY-09, oridonin; GSDMD cleavage inhibitor: Ac-FLTD-CMK [22]

**Fig. 2:**
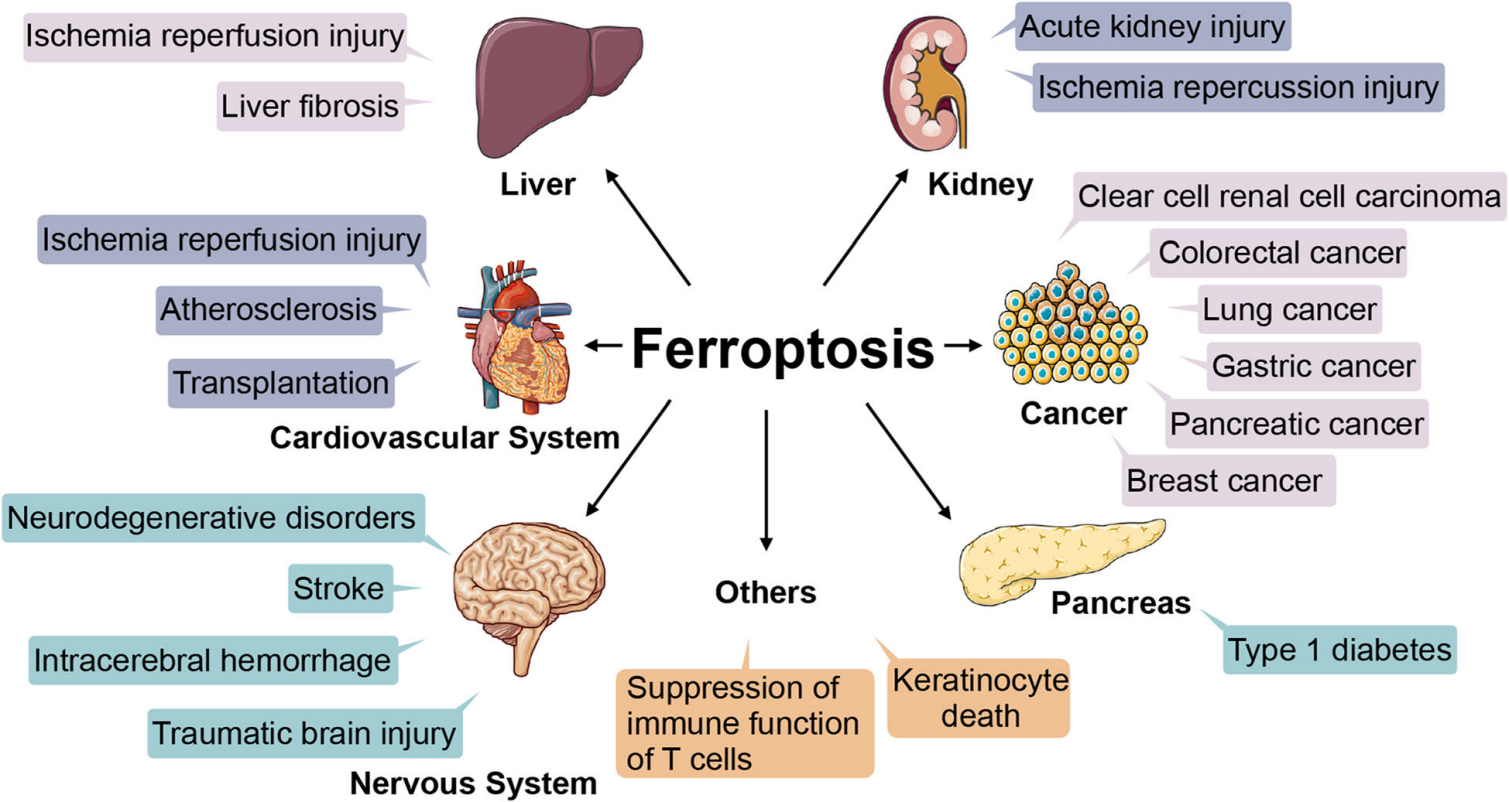
Ferroptosis involvement in pathological processes. Ferroptosis is involved in lesions of the liver, cardiovascular system, nervous system, pancreas and kidney. It is also associated with cancers and other pathological processes such as the suppression of immune function of T cells and keratinocyte death

Ferroptosis has been considered to be ﻿involved in multiple pathological processes according to current studies (shown in Fig. 2) [5, 72, 73]. For instance, it is linked to ischaemia reperfusion injury (IRI) in liver, heart and kidney and neurodegenerative diseases such as Alzheimer’s disease, Parkinson’s disease and Huntington’s disease [74, 75]. Other diseases such as liver fibrosis, stroke, type 1 diabetes, atherosclerosis and acute kidney injury are also associated with ferroptosis [8]. With the new concept of ferroptosis, our cognition of the mechanism of many diseases may be changed. Furthermore, ferroptosis is observed in various types of cancer, including breast [12], gastric [11], lung [76], and pancreatic cancer [9], among others, providing a novel method to treat malignancies [24].

## Current status of ferroptosis studies in breast cancer

Breast cancer can be divided into several types, including luminal A/B, HER-2 enriched, basal-like and normal-like subtypes. According to NCCN guidelines, endocrine therapy is used for ER-positive breast cancer and anti-HER2 targeted therapy is used for HER2-positive breast cancer. However, there is no targeted therapy for triple-negative breast cancer. Due to the lack of effective endocrine therapy and anti-HER2 targeted therapy, triple negative breast cancer patients are characterized by high recurrence rates and poor prognosis. Recently, some researchers have found that triple-negative breast cancer is more sensitive to ferroptosis than ER positive breast cancer [17]. Therefore, triggering ferroptotic cell death of breast cancer seems to be an effective treatment strategy, especially in triple negative subtype.

### Ferroptotic regulators in breast cancer

**Fig. 3:**
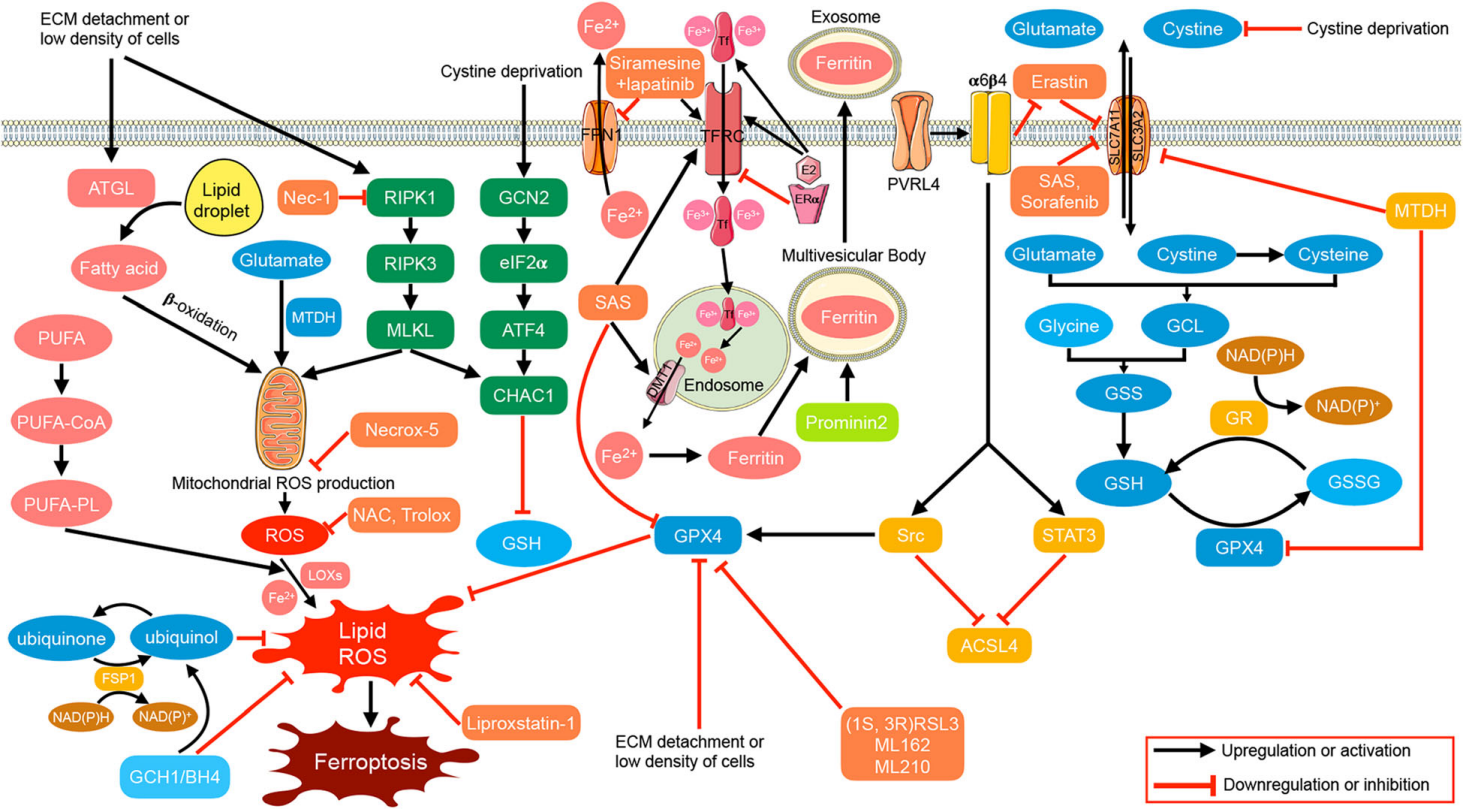
Published molecular mechanisms of ferroptosis in breast cancer. Based on the overview described in Fig. 1, some specific mechanisms are proposed in breast cancer. Low cell density triggers increased catabolism of neutral triglycerides from lipid droplets via ATGL to channel fatty acids to mitochondria for β-oxidation, producing ROS. A low cell density induces depletion of GSH via the RIPK1/RIPK3/MLKL/CHAC1 pathway. GSH is also inhibited by cystine deprivation via both a direct decrease in synthesis and the GCN2/eIF2α/ATF4/CHAC1 pathway. Activation of the RIPK1/RIPK3/MLKL pathway also induces mitochondrial fragmentation and ROS production, which could be suppressed by the mitochondrial ROS scavenger Necrox-5 and RIPK1 inhibitor Nec-1. Cellular ROS attack PUFA-PL to produce lipid ROS in the presence of LOXs and Fe^2+^, and the lipid ROS further induce ferroptosis. MTDH enhances the ability of cells to use intracellular glutamate to maintain respiratory chain activity. Cellular ROS can be reduced by NAC and trolox, and lipid ROS can be reduced by ubiquinol, GCH1/BH_4_, liproxstatin-1 and GPX4. GPX4 is inhibited by ECM detachment or a low density of cells, MTDH and inhibitors such as SAS, (1S, 3R) RSL3, ML162 and ML210. SAS also inhibits DMT1 and system xc-. The integrin α6β4 sustains GPX4 expression via Src and suppresses ACLS4 via Src and STAT3. Additionally, α6β4 attenuates the effect of erastin on xCT. The adhesion protein PVRL4 is necessary for α6β4 to exert its anti-ferroptotic function. The antiporter xCT is inhibited by erastin, SAS, sorafenib and MTDH. E2 upregulates expression of TFRC and secretion of transferrin, while ERα suppresses expression of TFRC. Administration of siramesine and lapatinib increases TFRC and decreases FPN1 expression, thus elevating the level of intracellular iron

Ferroptosis has been studied or implicated in researches of breast cancer. Here we reviewed ferroptosis-associated studies in breast cancer up to date, and presented main regulators and agents involved in Fig. 3.

Inhibition of GPX4 is a feature of ferroptosis as mentioned above. The ferroptotic agent (1S, 3R)-RSL3 and sulfasalazine (SAS) inactivate the peroxidase activity of GPX4 in breast cancer cells [23, 77]. Interestingly, drug-tolerant persister breast cancer cells acquire a dependency on GPX4, which means they are vulnerable to ferroptosis induced by GPX4 inhibition [78]. This result suggests an inhibition of GPX4 as a potential measure to overcome drug resistance in breast cancer. The normal function of GPX4 relies on the presence of glutathione (GSH). Starvation of cystine, a substrate used to synthesize GSH to prevent ferroptosis, was able to induce cell necroptosis and ferroptosis in TNBC via the GCN2-eIF2α-ATF4-CHAC1 pathway [79]. This process could be rescued by necrostatin-1 (Nec-1, a RIP1 inhibitor), necrosulfonamide (an MLKL inhibitor), deferoxamine (an iron chelator), ferrostatin-1 (a ferroptosis inhibitor), Necro-5 (a mitochondrial ROS scavenger) and RIP1 knockdown. The authors claimed that the stress response pathway but not apoptosis or autophagy-mediated cell death was involved. Notably, TNBC cell lines showed more sensitivity to cystine starvation and SAS than luminal cell lines in that study. Yu et al. also confirmed the efficacy of SAS for inducing ferroptosis in breast cancer cells by inhibiting expression of GPX4 and xCT, and upregulating the expression of transferrin receptor (TFRC) and divalent metal transporter 1 (DMT1), especially in cells with low oestrogen receptor (ER) expression [80].

The presence of relatively high levels of intracellular iron is required for ferroptosis. Ma et al. reported that lysosome-disrupting agents, siramesine and a tyrosine kinase inhibitor, lapatinib, synergistically induced cell death in breast cancer cells by disrupting cell iron metabolism and redox homeostasis. Lapatinib is an inhibitor of EGFR and HER2, but siramesine and lapatinib might induce ferroptosis by acting on transferrin and ferroportin, which transported iron into cells and exports iron out from cells respectively, other than on EGFR or HER2 [12]. In contrast, prominin2 could inhibit ferroptosis in breast cancer cells by promoting the formation of ferritin-containing multivesicular bodies (MVBs) and exosomes that transport iron out of the cell [81].

As lipid ROS is the main killer in ferroptosis, the role of lipid metabolism has been studied. Among the ACSLs, ACSL4 is specifically linked to ferroptosis, acylating PUFAs, especially arachidonic acid (AA) and adrenic acid (AdA), which are then inserted into membrane phospholipids by lysophosphatidylcholine acyltransferase 3 (LPCAT3) [34, 35]. In breast cancer, ACSL4 has been reported to be preferentially expressed in a panel of basal-like breast cancer cell lines and promote ferroptosis by enriching cellular membranes with long polyunsaturated n-6 fatty acids [17]. The author claimed that the position of the last double bond is more critical for induction of ferroptosis than the degree of unsaturation. Beatty et al. found that the conjugated linoleic acid (LA) α-eleostearic acid induced ferroptosis in TNBC, depending on acyl-CoA synthetase long-chain isoforms other than glutathione or GPX4 activity. In contrast to the previous study, the authors highlighted that three conjugated double bonds in the conjugated LA were required for ferroptotic activity, while their positioning and stereochemistry were less important [82].

The status of p53 in ferroptosis has been well established and broadly accepted [25, 26, 83], and its ferroptotic function in breast cancer has also been studied. In 2017, Xie et al. supported the positive regulatory role of p53 in erastin-induced ferroptosis in breast cancer cells, although p53 limited ferroptosis in colorectal cancer [24].

Interestingly, research has shown that ferroptosis can be regulated by cell adhesion and cell density in breast cancer. The α6β4 integrin, which had been shown to participant in cancer progression, contributes to the formation of cell adhesion [84, 85]. In 2017, Caitlin et al. showed that extracellular matrix (ECM) detachment is a physiologic trigger of ferroptosis in breast cancer cells due to suppression of GPX4, and α6β4 integrin can help cells evade this process by protecting against changes in membrane lipids, activating Src and STAT3 and suppressing expression of ACSL4 [86]. The α6β4 integrin not only ﻿eases ferroptosis triggered by ECM detachment but also protects adherent epithelial and carcinoma cells from ferroptosis induced by erastin. In the following year, they found that the PVRL4/α6β4/Src signalling pathway sustained GPX4 expression. In the absence of α6β4, PVRL4-mediated cell clustering induced an increase in lipid peroxidation, making cells sufficient for ferroptosis, while inhibition of both α6β4 and cell clustering made cells more susceptible to apoptosis than to ferroptosis [87]. A similar phenomenon was observed in a study by Panzilius et al., who found that ﻿low cell density sensitizes﻿ primary mammary epithelial and breast cancer cells to induction of ferroptosis by accumulating neutral triacylglycerides (TAG) enriched with PUFAs and triggering liberation of fatty acids from lipid droplets by adipose triglyceride lipase (ATGL) to fuel β-oxidation, whereas a high cell density confers resistance [88, 89]. In contrast to previous studies, this induction of ferroptosis is independent of the oncogenic pathway, cellular phenotype and expression of ACSL4.

Metadherin (MTDH), which was overexpressed and predicted a poor prognosis in cancer [90–92], conferred a therapy-resistant mesenchymal-high state in breast cancer cells, but also increased sensitivity to ferroptosis by downregulating GPX4 and SLC3A2. Moreover, MTDH enhanced the ability of breast cancer cells to use intracellular glutamate to maintain respiratory chain activity, which has been demonstrated to be an important metabolic process promoting ferroptosis [93, 94].

In 2020, Kraft et al. identified GTP cyclohydrolase 1/tetrahydrobiopterin as a suppressor of ferroptosis through synthesis of BH_4_/BH_2_ and lipid remodelling in cancer including breast cancer. The authors pointed out that this mechanism is independent of the GPX4/GSH system [95].

### Different susceptibilities to ferroptosis in diverse subtypes of breast cancer

Data have shown that basal-like breast cancers are more susceptible to ferroptosis, with mechanisms that remain to be explored [37, 79, 80, 93, 95]. According to published works, this susceptibility may be attributed to the reasons described below.

#### Expression of ERα and HER2

Stearoyl-CoA desaturase (SCD1, SCD), an enzyme that catalyses the rate-limiting step in monounsaturated fatty acid (MUFA) synthesis, has been shown to protect ovarian cancer cells from ferroptosis and regarded as a therapeutic target in ovarian cancer [96, 97]. Belkaid et al. illustrated that oestrogen induces SCD-1 expression and activity in ERα-positive breast carcinoma cells [98]. Thus, ERα may contribute to resistance to ferroptosis by upregulating SCD-1 in ERα-positive breast cancer. Additionally, ERα has been shown upregulate the level of nuclear factor E2-related factor 2 (NRF2) and downregulate that of Kelch-like ECH-associated protein 1 (KEAP1) [28]. In 2019, Yu et al. found that ERα could also confer resistance to ferroptosis by downregulating expression of TFRC [80]. Of note, the authors also showed a high expression level of TFRC in carcinoma tissues of patients with HER2+ breast cancer, but the regulatory machinery was not demonstrated in the study. In the study conducted by Doll et al., a HER2+ cell line (MDA-MB-453) and ER+ cell lines (T47D and MCF7) both showed resistance to ferroptosis induced by RSL3 or downregulation of FSP1 compared with triple-negative cell lines, but the ER+ and HER2+ cell line BT474 showed susceptibility similar to TNBC [37]. These results implied a complicated interaction between ER, HER2 and ferroptosis-associated pathways in breast cancer.

#### Metabolism

The sensitivity to ferroptosis is tightly linked to amino acid metabolism [72]. In 2013, Timmerman et al. constructed a functional metabolic portrait of 46 independently derived breast cell lines and found that nutrient preference varied widely among breast tumours [99]. Luminal-like cell lines preferred high glucose, while basal and claudin low TNBC cell lines showed more dependence on glutamine. Restriction of glutamine intake or inhibition of system xc^−^ activity increased the level of intracellular ROS and slowed the growth of TNBC. Glutamine is an important source of glutamate for supplying system xc^−^ activity to import cystine, which is essential for GSH synthesis [100]. The study conducted by Gao et al. also demonstrated the critical role of glutamine in ferroptosis [32]. Further gene expression enrichment analysis suggested enrichment of SLC7A11, CD44 and GCLM in TNBC [99]. SLC7A11 is a subunit of system xc^−^, and CD44 has been reported to regulate activity of system xc^−^ in breast cancer [101, 102]. Chen et al. verified the vital role of cystine in the survival of TNBC cells. Cystine starvation induced ferroptosis in TNBC by activating the GCN2-eIF2α-ATF4- CHAC1 pathway [79].

Sarmiento-Salinas et al. found a higher level of ROS in TNBC than ER+ breast cancer, and mitochondria were the main source of ROS in TNBC cell lines [103]. However, they pointed out that maintenance of a certain level of ROS is essential for survival of TNBC, and antioxidant administration could induce cell death. ROS regulates signalling pathways by altering the activity of target proteins such as PTP1b, PTEN, and MAPK phosphatases by forming modifications such as disulphide bonds (−SS-) and sulfenyl amide (−SN-) [104]. In contrast, Dong et al. showed that a relatively low level of ROS increased CSC-like properties in basal-like breast cancer by enhancing activity of β-catenin, and a decrease in ROS was induced by snail-mediated fructose-1,6-biphosphatase (FBP1) suppression [105]. In summary, characteristics of metabolism are different in subtypes of breast cancer, inducing different ROS levels and different susceptibilities to ferroptosis. Meticulous regulation of ROS determines the fate of cells.

### Development of ferroptotic therapeutic strategies

Researchers have attempted to apply ferroptosis to overcome therapy resistance in breast cancer (listed in Table 2). In 2017, Mai et al. demonstrated that ironomycin, a synthetic derivative of salinomycin, could kill breast cancer stem cells by accumulating and sequestering iron in lysosomes. When degradation of ferritin in lysosomes was triggered by cytoplasmic depletion of iron, the iron overload would produce ROS and promote ferroptosis [51].

**Table 2:** Targeting ferroptosis in breast cancer- currently available pharmaceutical agents

Drug	Origin	Improvement/function	Phenomenon and characteristics	Year	Reference
Ironomycin	Salinomycin	Accumulating and sequestering iron in lysosomes	Iron overload and reactive oxygen species overgeneration	2017	[51]
CSO-SS-Cy7-Hex/SPION/Srfn	Sorafenib, iron	Combined iron and sorafenib	Iron overload, intracellular depletion of glutathione and reactive oxygen species overgeneration	2019	[106]
Erastin@FA-exo	Erastin	TNBC targetting, increased biocompatibility	Intracellular depletion of glutathione and reactive oxygen species overgeneration	2019	[107]
MnO_2_@HMCu_2-x_S nanocomposites	Mn^2+^, rapamycin	Combined Fenton-like reaction by Mn^2+^ and autophagy by rapamycin	Iron overload and reactive oxygen species overgeneration	2019	[108]
Drug-organics-inorganics self-assembled nanosystem	Doxorubicin, iron, tannic acid	Combined chemotherapy, ferroptosis and superoxide dismutase (SOD)-like reaction	Iron overload and reactive oxygen species overgeneration	2019	[109]
Nanoparticle ferritin-bound erastin and rapamycin	Erastin, rapamycin	Combined ferroptosis and autophagy	GPX4 downregulation and lipid peroxidation accumulation	2019	[110]
Ascorbate plus nanocarrier loading Fe^3+^ and RSL3	Ascorbate, iron, RSL3	Increased specificity to target cancer cells by ascorbate	Iron overload, inhibition of GPX4 and reactive oxygen species overgeneration	2019	[111]

Sorafenib has been verified to induce ferroptosis in several kinds of cancer [112]. In 2019, to increase the efficacy of sorafenib, Sang et al. synthesized tumour-targeted and mitochondrial membrane anchored oxidation/reduction response and Fenton-Reaction-Accelerable magnetic NIR nanophotosensitizer micelles (CSO-SS-Cy7-Hex/SPION/Srfn). This complex increased the effect of sorafenib on killing cancer cells. It also provided good biosafety and effects of ferroptosis therapy in vivo, making it a promising candidate for clinical use in the future [106].

Yu et al. developed ﻿the targeted exosome-encapsulated erastin by labelling the exosomes with folate (FA) (erastin@FA-exo), which is overexpressed in TNBC. The erastin@FA-exo promoted ferroptosis with intracellular depletion of glutathione and ROS overgeneration and exhibited a better inhibitory effect on the proliferation and migration of MDA-MB-231 cells compared with erastin@exo and free erastin [107].

An et al. developed MnO_2_@HMCu_2-x_S nanocomposites (HMCMs), which possessed photothermal-enhanced glutathione depletion capability to induce inactivation of GPX4, and released Mn^2+^ to generate ROS by the Fenton-like reaction, thus inducing lipid hydroperoxide (LPO) accumulation and ferroptosis. Moreover, rapamycin, an autophagy promotor, was loaded into HMCM to reinforce ferroptosis via autophagy [108].

Xiong et al. developed a drug-organics-inorganics self-assembled nanosystem (DFTA), which combined chemotherapy, ferroptosis and photothermal therapy (PT), to improve ER+ breast cancer treatment. This nanosystem contained doxorubicin (DOX) as the chemotherapeutic agent, ferric chloride (FeCl_3_) as the ferroptosis inducer and tannic acid (TA) as the activator of the superoxide dismutase (SOD)-like reaction in the intracellular cascade. When treated with DFTA and a laser, intracellular GSH was significantly reduced through PT-mediated ROS production and ROS-produced intracellular oxidative stress cascade amplification, thus inducing ferroptosis [109].

Li et al. constructed a novel carrier-free nanodrug called nanoparticle ferritin-bound erastin and rapamycin (NFER), combining ferroptosis and autophagy in cancer treatment. NFER induced ferroptosis by GPX4 downregulation and lipid peroxidation accumulation, and the autophagy process induced by rapamycin in NFER also strengthened ferroptosis. This nanodrug exhibited potent anticancer activity in breast cancer cells in vitro and in vivo [110].

An et al. combined nanocarriers loading Fe^3+^ and RSL3 with ascorbate (Asc), which was reported to selectively kill cancer cells by accumulating hydrogen peroxide (H_2_O_2_) only in tumour extracellular fluids, to induce ferroptosis in cancer cells more accurately [111].

In conclusion, the portrait of ferroptosis has been outlined, characterized by three hallmarks as described above. The mechanisms of ferroptosis continue to be discovered in recent years, and targeting agents have been developed. However, as a newly coined cell death, more problems remain to be solved. For example, what is the marker of ferroptosis? How does lipid ROS kill cells? What is the relationship between ferroptosis and other types of cell death? We reviewed some studies in breast cancer to understand previous cell death from a new perspective and examine those regulators of ferroptosis from a historical perspective.

### Crosstalk between ferroptosis, apoptosis, necroptosis, autophagy-dependent cell death, and pyroptosis

Regulated cell death, as a normal life process, was first observed by Karl Vogt in toads in 1842 [22], but the scientific research of regulated cell death did not start until the proposal of “apoptosis” in 1972 by Kerr et al. To date, various patterns of regulated cell death have been found, and the interfaces between different death patterns are always attractive to researchers.

**Fig. 4:**
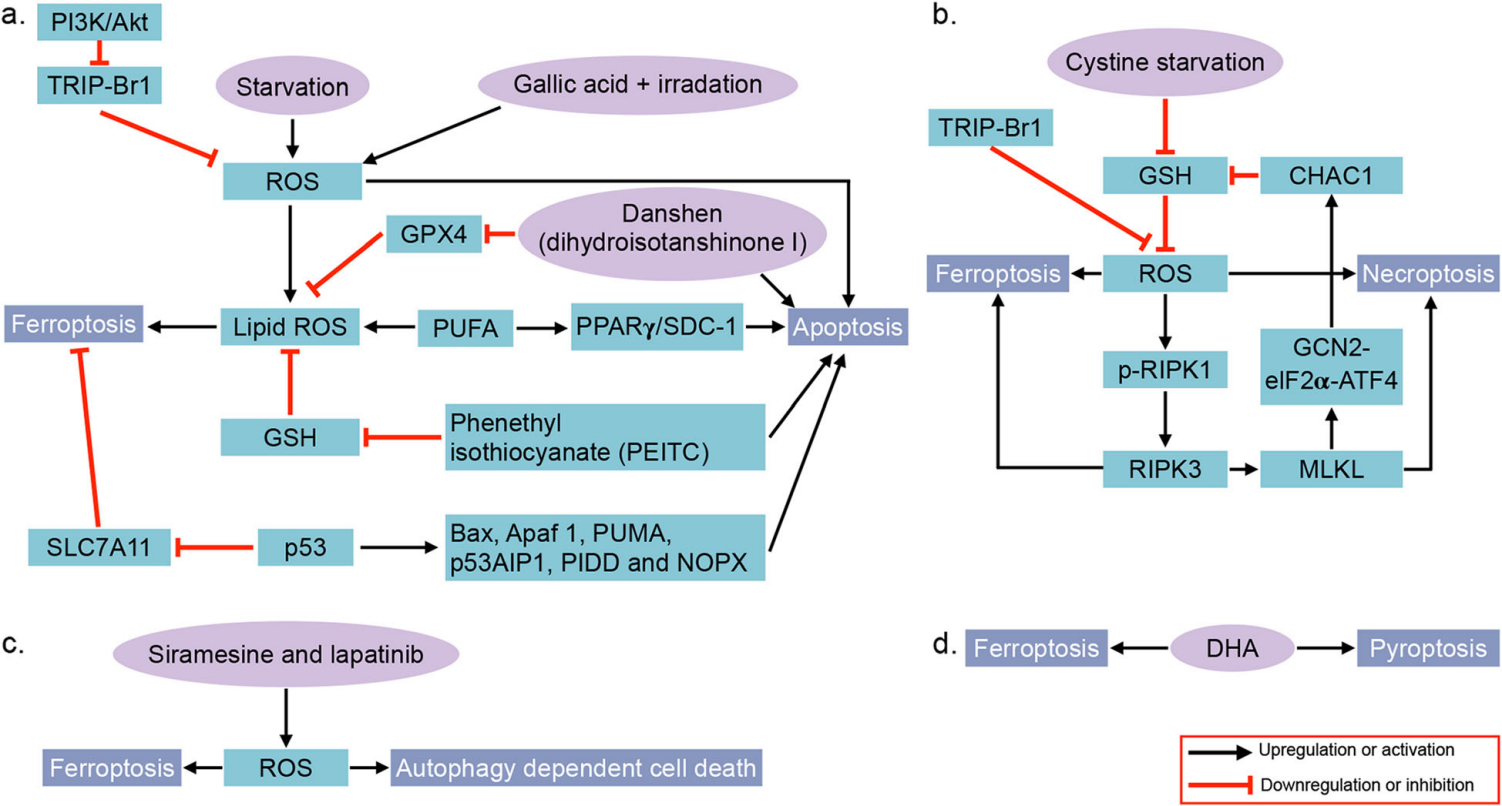
The crosstalk between ferroptosis and apoptosis, necroptosis, autophagy-dependent cell death and pyroptosis. a.The crosstalk between ferroptosis and apoptosis. Starvation and treatment of gallic acid and irradiation lead to upregulated ROS, and ROS in turn induces apoptosis and ferroptosis (via lipid ROS). ROS is decreased by TRIP-Br1, which is inhibited by the PI3K/Akt pathway. PUFAs induce ferroptosis in the form of lipid ROS, as well as apoptosis via the PPARγ/SDC-1 pathway. The lipid ROS is reduced by GPX4 and GSH, which are inhibited by DT in danshen and PEITC, respectively. DT and PEITC also induce apoptosis. P53 induces ferroptosis by inhibiting SLC7A11 and apoptosis by regulating genes such as BAX, APAF-1, PUMA, p53AIP1, PIDD and NOXA. b. Cystine starvation induces a decrease in GSH and subsequent increase in intracellular ROS, which triggers ferroptosis. The increased ROS also induces necroptosis via the RIPK1/RIPK3/MLKL pathway. TRIP-Br1 suppresses ferroptosis and necroptosis by inhibiting ROS production. In addition, MLKL upregulates CHAC1 via the GCN2/eIF2α/ATF4 pathway, and CHAC1 decreases the level of GSH. Upregulated RIPK3 causes breast cancer cells to rely on cystine and undergo necroptosis and ferroptosis upon cystine deprivation. c. Siramesine and lapatinib induce both ferroptosis and autophagy by upregulating cellular ROS. d DHA triggers both ferroptosis and pyroptosis in breast cancer cells.

As claimed by Stockwell, apoptosis has been consistently confused with cell death, especially in studies before the year 2000. In fact, multiple cell death modes can be triggered by the same stresses. However, in most cases, the cell death mode is not fully studied due to the lack of detection of multiple death pattern markers [113]. These cell death modalities are distinct but exhibit a considerable degree of interconnectivity [61]. Here, we compare ferroptosis with the other four common types of regulated cell death, i.e., apoptosis, necroptosis, autophagy-dependent cell death and pyroptosis. The associations between ferroptosis and these death patterns in breast cancer are illustrated as Fig. 4.

#### Apoptosis

As the most well-known regulated cell death, apoptosis was first coined in 1972 by Kerr, Wyllie, and Currie [114]. Studies have demonstrated some common pathways and key nodes between the “ancient” cell death and novel ferroptosis.

Apoptosis and ferroptosis can be induced by a common mechanism in breast cancer. As described above, PUFAs can induce ferroptosis in an ACSL4-dependent manner, and induce apoptosis via the peroxisome proliferator-activated receptor gamma (PPARγ)/SDC-1 pathway [82, 115]. Lipid peroxidation is the main killer in the process of ferroptosis, which could derive from lipids by being “attacked” by intracellular ROS [18]. ROS is also a potent driver of apoptosis in breast cancer [116], and multiple drugs induce apoptosis via ROS-mediated pathways [117–122]. Cystine starvation has been shown to induce ferroptosis in breast cancer cells as described above [79]. Additionally, starved breast cancer cells in glucose-, amino acid- and serum-free conditions can also induce apoptosis, autophagy and necroptosis, while the transcriptional regulator interacting with the PHD-bromodomain 1 (TRIP-Br1) inhibits cell death by stabilizing the X-linked inhibitor of apoptosis protein (XIAP) and decreasing ROS production [123]. P53, which has long been known as a regulator of apoptosis in cancers including breast cancer by controlling expression of apoptosis-associated genes such as BCL2-associated X protein (BAX), apoptotic peptidase activating factor 1 (APAF-1), p53-upregulated modulator of apoptosis (PUMA), p53-regulated apoptosis-inducing protein 1 (p53AIP1), p53-induced death domain protein 1 (PIDD) and Phorbol-12-Myristate-13-Acetate-Induced Protein 1 (NOXA) [124, 125], has been shown to participate in ferroptosis in breast cancer as mentioned above.

Some treatments induce both ferroptosis and apoptosis. For example, when treated with gallic acid and low-level laser irradiation, both apoptosis and ferroptosis were observed in a human non-tumourigenic breast epithelial cell line (MCF10A) and breast cancer cell line (MDA-MB-231) [126]. Danshen, a kind of Chinese traditional drug, has protective effects in breast cancer patients, which could be attributed to dihydroisotanshinone I (DT), a pure compound present in danshen, through inducing apoptosis and ferroptosis in breast cancer cells [127]. Phenethyl isothiocyanate (PEITC), a glutathione-depleting agent triggering upregulated ROS level and ferroptosis in several types of cancers, also induces apoptosis in breast cancer cells [128–131]. Thus, apoptosis and ferroptosis could co-exist in cells in response to similar stimulations and synergistically induce cell death.

Nevertheless, an opposite opinion in studies of other types of cells claimed that activation of caspase-3 and -8 requires a reducing environment maintained by intracellular glutathione [132, 133], which means GSH depletion in the process of ferroptosis may inhibit the normal occurrence of apoptosis [134]. Apoptosis and ferroptosis may co-exist and switch in different phases of cell death.

#### Necroptosis

Necroptosis is a form of programmed necrosis, which is regulated by receptor-interacting protein (RIP)1, RIP3, and mixed lineage kinase domain-like pseudokinase (MLKL) [135]. Studies in other diseases have shown a relationship between necroptosis and ferroptosis. In 2017, Müller et al. found that necroptosis and ferroptosis are alternative cell death pathways in acute kidney failure. Resistance to one pathway sensitized cells to death via the other pathway [136]. In 2018, Wu et al. found that 2-amino-5-chloro-N, 3-dimethylbenzamide (CDDO) inhibits both necroptosis and ferroptosis by inhibiting heat shock protein 90 (HSP90) [137], implying a common pathway between the two types of cell death. Another node connecting necrosis and ferroptosis is NADPH: the regulation of necrosis leads to the depletion of NADPH, and the lack of NADPH sensitizes cells to ferroptosis [21]. Tonnus et al. hypothesized that the free diffusion of NADPH between cells by tight junctions and gap junctions may contribute to spreading of necrosis and ferroptosis from one cell to the adjacent ones [138].

In breast cancer, Tang et al. found that cystine deprivation induced rapid programmed necrosis in breast cancer cells by activation of TNFα and MEKK4-p38-Noxa pathways. The results showed a correlation between addiction to cystine and a mesenchymal phenotype of cancer cells [139]. This phenomenon is very similar with that in ferroptosis. In the same year, Chen et al. found that cystine starvation induced both necroptosis and ferroptosis in TNBC via the GCN2-eIF2α-ATF4-CHAC1 pathway, implying a common mechanism regulating both necroptosis and ferroptosis in breast cancer [79].

Intracellular ROS have also been reported to be important inducers of necroptosis [140–142]. Numerous drugs induce necroptosis via ROS in breast cancer [143–145]. In 2015, Jung et al. found that TRIP-Br1 could confer resistance to serum starvation-induced cell death including necroptosis by suppressing cellular ROS production in breast cancer cells [123]. RIPK3, a key effector in programmed necrotic cell death, was found to be overexpressed in recurrent breast tumour cells from a murine model of breast cancer recurrence. The upregulated expression of RIPK3 caused the recurrent tumour cells to rely on extracellular cystine and undergo necroptosis and ferroptosis upon cystine deprivation with erastin [146].

#### Autophagy-dependent cell death

The relationship between autophagy and ferroptosis has been well examined in our previous review [147]. Autophagy-dependent cell death is a type of cell death triggered by a high level of cellular autophagy, as termed by Levine et al. in 2013 [148]. Studies have demonstrated that autophagy can promote ferroptosis by providing iron via the degradation of ferritin, termed ferritinophagy, in cancer cells, mediated by nuclear receptor coactivator 4 (NCOA4) [134, 149]. Gao et al. applied RNAi screening coupled with subsequent genetic analysis and identified multiple autophagy-related genes including NCOA4 as positive regulators of ferroptosis. Due to the association between autophagy and ferroptosis, the authors concluded that ferroptosis is an autophagic cell death process [33]. Wu et al. found that levels of lysosome-associated membrane protein 2a were upregulated by a ferroptosis inducer erastin and subsequently promoted chaperone-mediated autophagy (CMA), which promoted the GPX4 degradation [137]. Additionally, chelation of redox-active iron, lysosomal activity, p53 modulation, and the p62-Keap1-NRF2 pathway may be involved in the interaction between autophagy and ferroptosis [147].

In breast cancer, autophagy-dependent cell death and ferroptosis could co-exist and alternately dominate cell death in a time-dependent manner. For example, in the study conducted by Ma et al., siramesine and lapatinib induced breast cancer cell death mainly by ferroptosis initially but switched to autophagy at 24 h. Ferroptosis and autophagy are both mediated by iron-dependent ROS generation, and degradation of ferritin by autophagy could continuously provide iron throughout the whole process [150]. ROS is an important mediator of autophagy in breast cancer [120, 121, 151], implying a role of ROS as a node between autophagy-dependent cell death and ferroptosis.

#### Pyroptosis

Pyroptosis is a form of regulated cell death triggered by caspase1, caspase4/5 (caspase1 and caspase11 in mice) and gasdermin D. Kang et al. demonstrated that GSDMD-induced pyroptosis was driven by lipid peroxidation in lethal polymicrobial sepsis. GPX4 could negatively regulate pyroptosis in macrophages, and the antioxidant vitamin E, which reduced lipid peroxidation, prevented polymicrobial sepsis in Gpx4^−/−^ mice [152].

Due to limited studies of pyroptosis conducted in breast cancer, crosstalk between pyroptosis and ferroptosis is rarely referenced. Limited results have shown that the n-3 PUFA docosahexaenoic acid (DHA), which was shown to induce ferroptosis in cancer [153, 154], also triggers pyroptosis in TNBC cells, implying that lipid metabolism might be an intersection point [155]. Since impairment of the plasma membrane is a vital process in both types of cell death, more common mechanisms remain of interest for exploration.

## Historical review of critical ferroptosis regulators in breast cancer

Based on current studies, iron, PUFAs, NRF2, GPX4, ACSL4, p53 and SLC7A11 have been identified as critical regulators of ferroptosis. Thus, we reviewed their roles in breast cancer in previous studies.

### Iron

#### Iron in ferroptosis

**Fig. 5:**
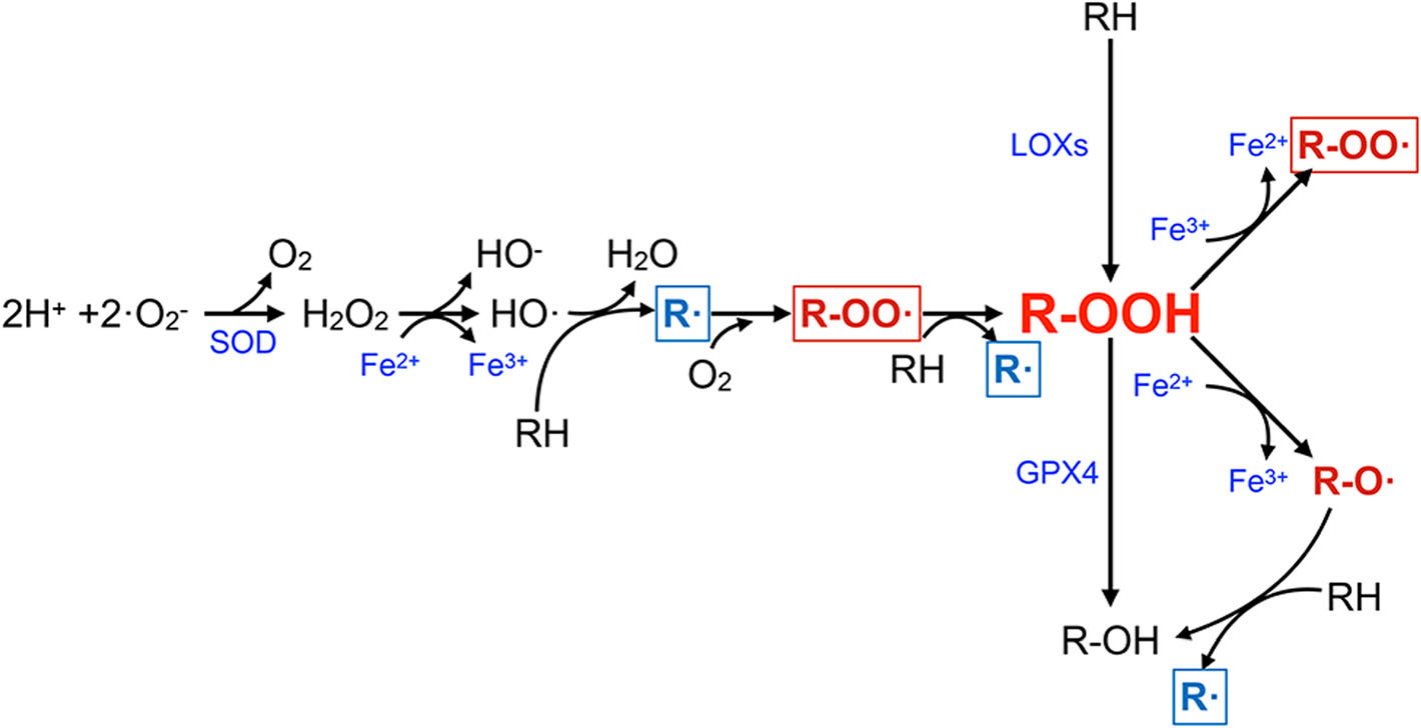
The role of iron in the generation of cellular oxygen radicals and lipid peroxidation. Superoxide dismutase (SOD) catalyses the dismutation of the superoxide (O_2_-) radical into oxygen (O_2_) and hydrogen peroxide (H_2_O_2_). Ferrous iron (Fe^2+^) is oxidized to ferric iron (Fe^3+^) through reaction with hydrogen peroxide (H_2_O_2_), to produce highly reactive hydroxyl radicals (OH·), which is termed the Fenton reaction. RH (refer to PUFA-PL) is attacked by HO· and forms R·, which then react with O_2_ into R-OO·. R-OO· reacts with another RH to produce R· and R-OOH. R-OOH is an oxidation product, which can also be derived from RH catalysed by LOXs. GPX4, the vital anti-ferroptotic factor, reduces R-OOH into nontoxic R-OH. In the presence of Fe^3+^ or Fe^2+^, R-OOH forms R-OO· or R-O·, respectively. The latter reacts with another RH to produce R-OH and R·. In this process, R· (labelled by a blue frame) and R-OO· (labelled by a red frame) are continuously generated and contribute to the amplification of oxygen radicals and lipid peroxidation production

As its name implies, execution of ferroptosis requires the existence of high levels of intracellular iron [4]. Ferroptotic death could be suppressed by iron chelators and promoted by transferrin and its receptor [14, 32, 156]. Ferritin, the intracellular iron storing protein, releases iron to promote ferroptosis when degraded by autophagy [16, 157]. Iron participates in ferroptosis due to its capacity to produce lipid ROS in an auto-amplifying manner (shown in Fig. 5) [158, 159].

#### Iron in breast cancer

Iron has a critical role in cellular energy producing and intermediate metabolism, owing to its ability to pass electrons by converting between ferric (Fe^3+^) and ferrous (Fe^2+^) oxidation states [160]. An iron enzyme is also involved in the synthesis of deoxyribonucleotides, ribonucleotide reductase, which is essential for cell proliferation [161]. As a result, iron is fundamental for cell survival, proliferation and differentiation [162]. Since iron plays such roles in cell growth and death, it is not surprising that iron intake and levels in breast tissue are associated with the risk of breast cancer. However, epidemiological studies have shown a controversial relationship between iron intake and breast cancer risk. Some research supports a positive relationship [163, 164], while others do not [165–169].

#### Iron promotes breast cancer progression

Levels of serum ferritin, a main form of iron in the human body, are higher in patients with breast cancer than the normal population [170, 171]. Studies suggested that tissue ferritin probably has more diagnostic value than serum ferritin. The ferritin concentration is 6-fold higher in breast cancer than benign tissues and predicts a bad prognosis [172–175]. Rossiello et al. indicated that high expression of ferritin in carcinoma tissue is mainly attributed to stroma inflammatory cells other than cancer cells [176]. In 2013, Alkhateeb et al. observed decreased ferritin expression in breast cancer cells but increased infiltration of ferritin-rich CD68-positive macrophages with increased tumour histological grade, and they showed that both apo- and holo-ferritin in the microenvironment promoted the proliferation of breast cancer cells without altering intracellular iron [177].

In vitro studies have also provided supporting evidence. Growth of the breast cancer cells is shown to be dependent on iron and transferrin in the culture medium [178, 179]. In 2009, Kim et al. showed that 15-Deoxy-Δ12,14-prostaglandin J2 (15d-PGJ2), an endogenous ligand of PPARγ, could upregulate the expression of haem oxygenase-1 (HO-1), level of ROS and subsequently expression of matrix metalloproteinase-1 in human breast cancer cells, and increased metastasis and invasiveness. This process could be inhibited by an iron chelator, implying the potential of iron to regulate intracellular ROS and related signalling pathways [180]. The iron chelator Dp44mT also exhibited anticancer effects in breast cancer [181].

Transferrin, TFRC and SLC11A2, which are responsible for importing iron into cells, are also expressed at higher levels in breast carcinoma than normal tissues, while the iron exporter SLC4A1 exhibited lower expression [176, 182, 183]. Expression of TFRC regulated intracellular total iron and proliferation and invasion of the breast cancer cells in vitro and in vivo [183]. In 2010, Shpyleva et al. observed an increased expression of ferritin light chain, ferritin heavy chain, transferrin, transferring receptor, and iron-regulatory proteins 1 and 2 in mesenchymal phenotype breast cancer cells than in the epithelial phenotype. Additionally, downregulation of ferritin with miR-200b sensitized MDA-MB-231 cells to the chemotherapeutic agent doxorubicin, implying that mesenchymal phenotype breast cancer cells were more dependent on iron metabolism and iron metabolism regulated sensitivity to chemotherapeutic drugs [184]. In 2015, Schneider et al. found that a vacuolaor-ATPase inhibitor archazolid could disrupt endocytic TFRC recycling in breast cancer cells, resulting in cellular iron depletion, which caused stabilization of the HIF1α protein, reduction of ribonucleotide reductase (RNR) activity and subsequently apoptosis in vitro and reduction of tumour growth in vivo [185]. These results implied an essential role of iron in normal metabolism of breast cancer cells.

#### Iron deficiency contributes to cancer cell survival, growth and metastasis

In 1986, Doroshow found that doxorubicin-induced cell death in the breast cancer cell line MCF7 could be prevented by oxygen radical scavengers and iron chelators [186]. A study conducted by Jian et al. showed that mice fed an iron-low diet had significantly higher tumour volumes and lung metastasis rates than those fed iron-high diets. Mechanistically, iron deficiency activated the Notch pathway and triggered EMT in a mouse model of triple negative breast cancer. It was also associated with lymph node invasion in young BC patients [187]. In 2015, Buranrat et al. found that both externally applied and endogenous ferritin could abate the toxicity of doxorubicin in breast cancer cells by decreasing doxorubicin-induced ROS. Ferritin may protect cancer cells from ROS by reducing free iron levels. The authors did not relate this phenomenon to ferroptosis, although the concept of ferroptosis has been coined for 3 years [188].

In summary, iron is essential for cellular normal metabolism and distributed at higher levels in tumour tissues, especially in tumours with high malignancy. It has been crucially involved in the production of ROS, and ROS is both an important promoter of cell proliferation and mediator of cell death in breast cancer, suggesting a vital role of a delicate balance of both iron and intracellular ROS levels in cancer cell [189–191]. Reasonably, cancer cells with higher intracellular iron and ROS are more dependent on the antioxidant system to maintain the balance.

#### Oestrogen and iron metabolism in breast cancer

Oestrogen is an important factor that induces breast cancer [192]; however, oestrogen alone cannot explain the rate differences of breast cancer recurrence and incidence between pre- and postmenopausal women. For example, the oestrogen level is lower in postmenopausal women, but the incidence of low-grade ER+ BC is higher [193]. Huang et al. partly attributed this phenomenon to the difference in iron load between pre- and postmenopausal women. Iron status, including serum (S-) ferritin and haemoglobin (Hb), could be influenced by age and menstrual bleeding, causing premenopausal women to be in an iron-deficient state and postmenopausal women in an iron overload state. Iron deficiency leads to overexpression of VEGF and HIF-1α, while iron overload induces activation of the MAPK pathway, resulting in increased breast cancer recurrence and incidence rates, respectively [194]. This conclusion is further supported by other researches [195, 196].

Current evidence implies that iron and oestrogen may act synergistically [197]. Oestrogen metabolites release Fe^2+^ from ferritin, which in turn generates hydroxyl radicals and contribute to the initiation of breast cancer [198]. Although serum oestrogen levels decrease after menopause, the 17β-oestradiol concentration in breast tissues does not change significantly [197]. The 17β-oestradiol can upregulate TFRC in ER+ breast cancer cells, and a high-17β-oestradiol and high-iron environment can promote the proliferation of breast cancer cells [199]. The 17β-oestradiol can stimulate hormone-responsive cells to secrete transferrin, while anti-oestrogen 4-hydroxy tamoxifen reduces secretion. This autocrine may help breast cancer cells acquire the ability to proliferate [200].

### Polyunsaturated fatty acids

#### PUFAs in ferroptosis

**Fig. 6:**
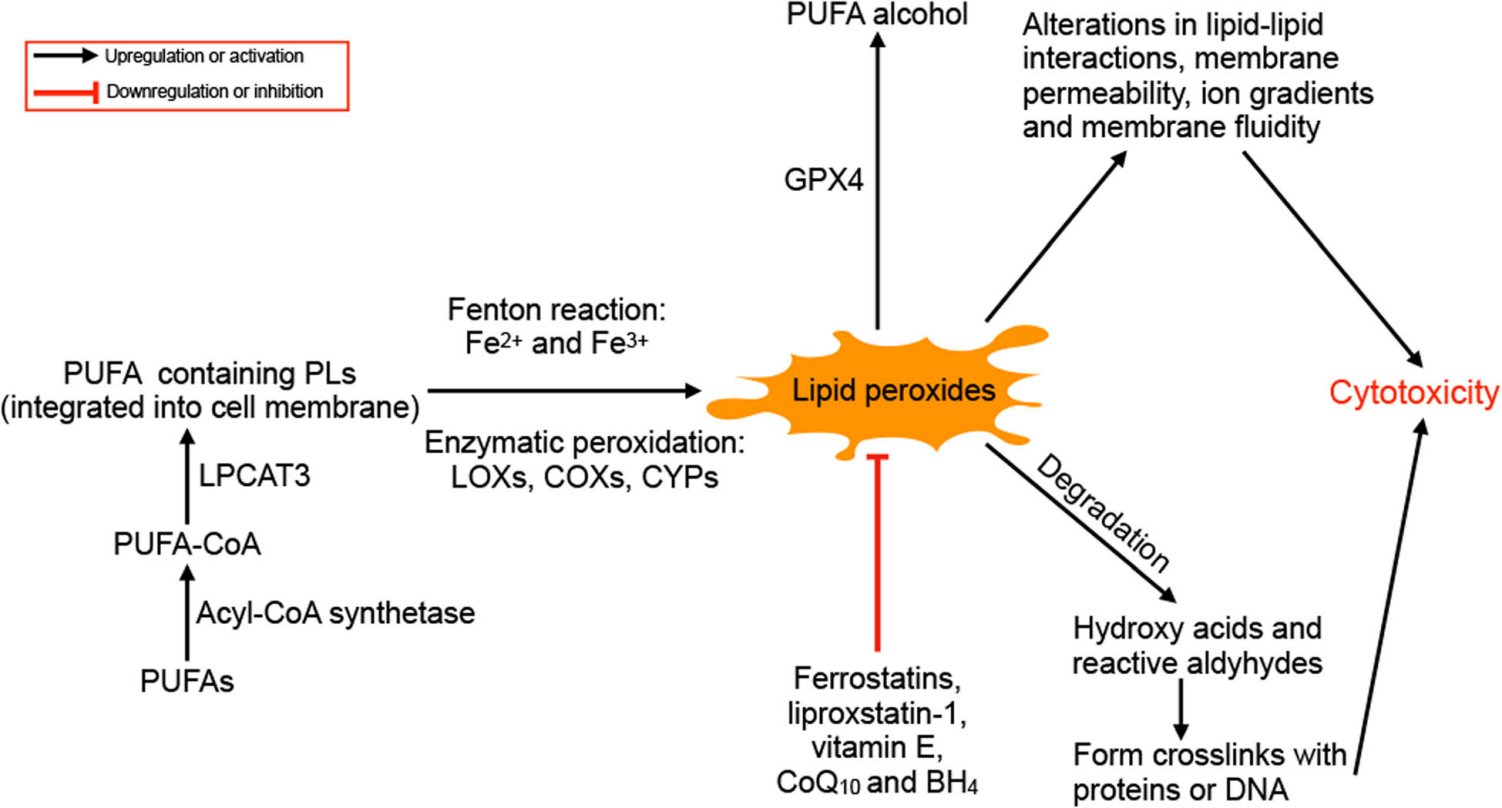
The role of PUFAs in ferroptosis. PUFAs are acetylated by acyl-CoA synthetase and integrated into phospholipids (LPs). The PUFAs containing PLs are oxidized through the non-enzymatic Fenton reaction or enzymatic peroxidation by LOXs, COXs and CYPs. The peroxidation of PUFAs can be inhibited by ferrostatins, liproxstatins, vitamin E, CoQ_10_ and BH_4_, and lipid peroxides can be reduced to the corresponding alcohol by GPX4. The lipid peroxides exert cytotoxicity by altering lipid-lipid interactions, membrane permeability, ion gradients and membrane fluidity, and their degradation products (mainly hydroxy acids and reactive aldehydes) can from crosslinks with proteins or DNA

The accumulation of lipid peroxidation products is considered to be the main killer in the ferroptosis, but the drivers of lipid peroxidation, subcellular location of lethal lipid peroxides and exact processes by which lipid peroxidation leads to cell death remain unsolved [113]. In 2015, the importance of the metabolism of PUFA-containing phospholipids (PLs) was highlighted in ferroptosis [34]. Dixon et al. identified nine genes involved in small-molecule-induced nonapoptotic cell death in KBM7 cells, including BAX, NOXA, AGPAT3, HSD17B11, LPCAT3, ACSL4, SETD1B, NADK, TECR, ACACA and ZDHHC5, among which ACSL4 and LPCAT3 were mediators of fatty acid metabolism. They also found a novel small molecular drug, CIL56, which triggered ferroptosis in an ACC1, a lipid synthetic enzyme, dependent manner [34]. PUFAs are highly susceptible to free radical or enzyme-mediated oxidation due to the presence of bis-allylic hydrogen atoms, which differs from saturated fatty acids (SFAs) and MUFAs [18]. PUFAs promote ferroptosis only when they are activated and incorporated into membrane PLs and become deadly when oxidized [3, 201]. The peroxidation of PUFAs at the bis-allylic position is catalysed by lipoxygenases and the Fenton action mediated by iron [158, 202]. Lipid peroxides are generally considered to exert their toxic effects by altering the structure of lipid membranes and crosslinking DNA and proteins via their metabolites (shown in Fig. 6) [18]. In 2018, Agmon et al. developed molecular dynamics models of lipid membranes and proposed a hypothesis that during ferroptosis, membrane thinning and increased curvature drives increased accessibility to oxidants and ultimately micellization, resulting in irreversible damage to membrane integrity [203].

#### PUFAs in breast cancer

The role of PUFA intake in the risk of breast cancer is controversial. A study by Löf et al. showed that PUFA reduced breast cancer risk among females over the age of 50 [204], while a meta-analysis by Cao et al. suggested no relationship between PUFAs and breast cancer [205]. PUFAs consist of n-3 PUFAs and n-6 PUFAs, according to the double-bond position, and researches have implied that PUFAs containing different double-bond positions have various effects on breast cancer risk [206, 207]. High intake of n-6 PUFAs is related to an increased risk of breast cancer [208–210], while n-3 PUFAs are protective factors factors [208, 210–212]. Chajès et al. found that increased n-3 PUFA intake is associated with a decreased risk of breast cancer in obese women (OR = 0.58, 95% CI = 0.39–0.87; *P* = 0.008), but not in normal weight or overweight women (Heterogeneity = 0.017), and the author attributed the difference to decreased inflammation and improved adipokine and oestrogen levels induced by n-3 PUFAs in adipose tissue in obese women [209]. Conversely, some research has also shown a non-significant relationship between breast cancer and n-3 or n-6 PUFAs [213–218]. The ratio of n-6/n-3 PUFAs may be more important for breast cancer risk than individual dietary amounts of these fatty acids [217, 219, 220].

#### Effect of PUFAs on breast cancer cells

The effect of PUFAs on breast cancer cells depends on various of factors, such as the concentration, degree of unsaturation, position of double bonds, types of cells and even density of cells. In 1979, Wicha et al. found a promoting effect of unsaturated free fatty acid at a low concentration and an inhibiting effect at high on the growth of normal and neoplastic rat mammary epithelial cells [221]. In the following years, a similar cytotoxicity of PUFAs in breast cancer cells was also reported in other studies [222–225]. In 1986, Begin et al. found that PUFAs could differentially induce cytotoxic effects depending on the number of double bonds in the fatty acid, cell density, fatty acid concentration, and type of cell. The most selective cytotoxic effects were determined with fatty acids containing 3, 4, and 5 double bonds, and PUFA-induced cell death increased as the PUFA concentration increased. Cancer cells, including breast cancer cells, were more susceptible to PUFAs than normal cells, and they gained some resistance when they were grown at a high density. Of note, selenium in the form of selenous acid could inhibit cytotoxic activity in a dose-dependent manner, suggesting that PUFA-induced cell death, which was determined by trypan blue in this study, might be ferroptosis [226]. In 2008, Sun et al. found that n-3 PUFAs induced breast cancer cell death by peroxisome proliferator-activated receptor γ-mediated upregulation of syndecan-1 [115]. In 2015, α-linolenic acid (ALA), a kind of n-3 PUFA, was shown to reduce growth of both-triple negative and luminal breast cancer in both high and low oestrogen environments, and MCF-7 luminal breast cancer cells exhibited relative resistance to ALA-induced cell death [227].

#### PUFAs exhibit antitumour activity by upregulating cellular ROS in breast cancer cells

PUFAs induce ferroptosis in the form of lipid ROS. Evidence has shown that administration of PUFAs elevates the level of intracellular ROS in breast cancer. In 1995, Shao et al. found that dietary menhaden oil enhanced mitomycin C antitumour activity in human mammary carcinoma by increasing lipid peroxidation, protein oxidation and the degree of fatty acid unsaturation in tumour membrane phospholipids, linking the anticancer effect of PUFAs to cellular ROS [228]. In the same year, Chajès et al. tested various PUFAs in breast cancer cells and demonstrated that the n-3 PUFAs ALA, DHA and eicosapentaenoic acid (EPA) arrested the growth of breast cancer cells except MCF7, and n-9 MUFA oleic acid (OA) stimulated proliferation of hormone-independent but not hormone-dependent cells, and the effect of n-6 PUFA γ-linoleic acid (GLA) varied in different cell lines. Of note, PUFA administration increased the cellular level of lipid hydroperoxides, and this level was positively related to the content of conjugated dienes in the added PUFAs. For example, DHA had the highest content of conjugated dienes, thus inducing the highest level of lipid hydroperoxides. Vitamin E could rescue the increased lipid hydroperoxides and suppression of proliferation induced by PUFAs, implying a vital role of lipid ROS in the cytotoxicity of PUFAs [229]. In contrast, Chamras et al. observed inhibition of ER+ breast cancer cell (MCF-7) proliferation induced by PUFAs but no changes in ROS levels, and the inhibition could not be significantly released by vitamin E. They also found no apoptosis or cell cycle alteration following incubation with PUFAs, suggesting a relative resistance of PUFA-induced ROS in ER+ breast cancer cells [230]. In 2008, Siddiqui et al. reviewed the mechanisms by which oxidation products of DHA induce cell death, and divided DHA oxidation into enzymatic processes by cyclooxygenases (COX), lipoxygenases (LOX) and cytochrome P450 monooxygenases (CYP) and non-enzymatic processes by free radical [231]. Lipid peroxidation induced a decrease in DHA containing cardiolipin (CL) and mitochondrial membrane potential, which in turn initiated apoptosis. Moreover, DHA could regulate apoptosis by activating nuclear peroxisome proliferator-activated receptor (PPAR) and retinoid X receptor (RXR). In the same year, Sun et al. verified that DHA promoted apoptosis in breast cancer cells by upregulating syndecan-1 via peroxisome proliferator-activated receptor-γ [115]. In 2017, Tsai et al. confirmed that DHA-induced cell death in breast cancer cells might be associated with increased ROS-mediated activation of the PI3k/Akt/Nrf2 pathway and upregulation of oxidative stress-induced growth inhibitor 1 (OSGIN1) [232].

The anti-cancer effect of PUFAs in breast cancer was also verified in animal models. In 1996, Rose et al. proved that the dietary n-3 PUFAs EPA and DHA inhibited lung metastases of breast cancer after surgical excision in nude mice [233]. In 1997, Noguchi reported that low-dose EPA and DHA reduced the incidence of breast cancer in rats induced by 7,12-dimethylbenz(a) anthracene (DMBA) [234]. In 1999, Rose and Connolly et al. found that reduced dietary LA (n-6 PUFA) intake and addition of dietary DHA (n-3 PUFA) inhibited the growth of breast cancer cell (MDA-MB-231) xenografts and induced apoptosis in nude mice [235]. The authors attributed this phenomenon to the reduction of prostaglandin E2 and 12- and 15-hydroxyeicosatetraenoic acids, which in turn reduced paracrine stimulation of microvessel endothelial cells and induced antiangiogenicity in nude mice [236].

#### PUFA-induced ROS sensitizes breast cancer cells to chemotherapy

The ROS-generating properties of PUFAs were applied to sensitize breast cancer to treatments. In 1998, Germain et al. proved that DHA and oxidant agents increased doxorubicin cytotoxicity in TNBC cells by increasing cellular levels of lipid peroxidation, and this effect could be abolished by the lipid peroxidation inhibitor dl-α-tocopherol [237]. In 2004, Colas et al. found that DHA sensitized rat autochthonous mammary tumours to radio treatment, and this effect was inhibited by vitamin E [238]. In 2008, Vibet et al. found that DHA sensitized MDA-MB-231 TNBC cells other than luminal MCF7 to anthracyclines, and the ROS level increased in MDA-MB-231 but not MCF7. The authors attributed the sensitization to a reduction of GPX1 induced by DHA. Vitamin E abolished the effect of DHA both during sensitization to chemotherapy and GPX1 inhibition [239]. Similarly, in 2009, Colas et al. demonstrated that addition of an antioxidant to the diet could suppress mammary tumour sensitivity to anthracyclines in fish oil-fed rats, and addition of an oxidant system acted in an opposite manner [240]. Kang et al. found that DHA induced cell death via ROS generation and caspase 8 activation in MCF7, while MDA-MB-453 and MDA-MB-231 showed resistance. They also showed that a fish oil diet increased intratumour levels of EPA and DHA in nude mice and suppressed breast cancer growth in vivo [241]. In 2018, Zhu et al. reported a synergistic effect of n-3 PUFAs and rapamycin (Rp) in breast cancer cell cycle arrest, apoptosis and autophagy blockage in vitro and in vivo. They noticed that combined treatment of n-3 PUFAs and Rp significantly inhibited glycolysis and glutamine metabolism, and the antitumour effects of this combination treatment were dependent on β-oxidation and oxidative phosphorylation-mediated ROS production [242].

Overall, ROS generation is a vital process in PUFA-induced cell death and seems to be affected by ER expression in breast cancer cells.

#### PUFA functions via mechanisms other than ROS generation

PUFAs also suppress breast cancer by regulating intracellular pathways and altering the cell membrane composition.

From 2001 to 2006, Menendez et al. conducted a series of studies confirming that MUFA OA and PUFAs including ALA, GLA, and DHA sensitize breast cancer cells to docetaxel, paclitaxel, vinorelbine or trastuzumab by decreasing the expression of HER2 [243–247]. In 2013, Zou et al. also verified that n-3 PUFAs prevented breast cancer by inhibiting the HER2 pathway in fat-1 transgenic mice, in which n-3 PUFA could be endogenously synthesized from n-6 PUFAs [248]. Furthermore, GLA was also shown to sensitize breast cancer cells to anticancer drugs such as paclitaxel, vinorelbine and docetaxel in a ROS-independent manner [244, 249, 250].

PUFAs can function by interacting with G protein-coupled receptors. In 2005, Sauer et al. showed that plasma EPA suppressed cell proliferation in MCF-7 human breast cancer xenografts via a G protein-coupled, n-3 PUFA receptor-mediated signal transduction pathway, which was sensitive to pertussis toxin [251]. In 2012, Cao et al. found that 17β-oestradiol enhanced inhibition of n-3 fatty acids of the growth of ER+ breast cancer cells independent of ERα. This effect was mediated by activation of the G protein coupled oestrogen receptor 1 (GPER1)-cAMP-PKA pathway and suppression of EGFR, Erk1/2 and AKT [252].

As cell membrane components, PUFAs inhibit the function of membrane proteins by modifying lipid rafts. In 2007, Schley et al. found that the n-3 PUFAs DHA and EPA could alter the composition of rafts on cell membranes and normal EGFR and MAPK signalling, thus inducing apoptosis in breast cancer cells [253]. In 2011, Corsetto et al. found that n-3 PUFA treatment could increase the degree of fatty acid unsaturation of cell membrane, reduce the activation of EGFR and induce cell death in breast cancer cells [254]. In 2012, Corsetto et al. demonstrated that n-3 PUFAs could decrease breast cancer cell proliferation by modifying lipid raft biochemical and biophysical features [255].

PUFAs also suppress breast cancer cell proliferation and induce cell death by regulating Bcl-2, p53, neutral sphingomyelinase (N-SMYase), EZH2 and transient receptor potential canonical (TRPC) 3 [256–259].

### NRF2

#### NRF2 in ferroptosis

NRF2 regulates the expression of many genes responsible for preventing lipid peroxidation in ferroptosis, and it is regarded as a therapeutic target in cancer [260, 261]. Sun et al. identified NRF2 as a negative regulator of ferroptosis by activating transcription of quinone oxidoreductase 1 (NQO1), HO-1, and ferritin heavy chain 1 (FTH1). P62 could enhance this process by preventing NRF2 degradation and increasing subsequent NRF2 nuclear accumulation through inhibiting KEAP1, which is a key regulator of cellular oxidative stress and tumour development [29, 262].

#### NRF2 in breast cancer

In breast cancer, an immunolocalization study identified NRF2 as an independent adverse prognostic factor for both recurrence and disease-free survival of patients, and its expression level was significantly associated with the histological grade, Ki-67 labelling index, p62 immunoreactivity, and NAD(P)H: NQO1 level [263]. In vitro, NRF2 was also shown to promotes cell proliferation and metastasis [264–266].

The role of NRF2 in antagonizing intracellular ROS in breast cancer cells has been broadly studied. In 1997, NRF2 was shown to regulate antioxidant responsive element (ARE), inducing expression of NAD(P)H, NQO-1 and glutathione S-transferase (GST) [267]. When suffering from oxidative stress, NRF2 upregulated the expression and activity of xCT in breast cancer cells to promote survival [268]. When ROS levels is reduced, the expression of NRF2 is also downregulated [269].

NRF2 could promote breast cancer cells to survive from drugs and other treatment by antagonizing ROS. In 2008, Kim et al. found an elevated expression of NRF2 in the tamoxifen-resistant MCF7 cell line. Additionally, the NRF2/ARE is critical for the enhanced expression of antioxidant proteins in TAM-resistant breast cancer cells [270]. In 2014, PERK-NRF2 signalling was found to protect breast cancer cells from chemotherapy by reducing ROS levels and increasing drug efflux [271]. Phenethyl isothiocyanate (PEITC), a naturally occurring electrophile, kills breast cancer cells by depleting intracellular GSH levels and triggering accumulation of ROS, while NRF2 confers cell resistance to PEITC by raising intracellular levels of GSH [130]. In 2016, Syu et al. showed that NRF2 could confer chemoresistance to breast cancer cells (MCF7) by eliminating ROS via the NRF2-GCLC-GSH pathway [272]. NRF2 could also enhance resistance to other treatment in breast cancer [273–276].

Nrf2 is targeted by KEAP1 for ubiquitination and proteasome-mediated degradation. The oxidative stress-mediated dipeptidyl-peptidase 3 (DPP3) -KEAP1 interaction enhanced the function of NRF2 to promote breast cancer cell survival [266]. Mammalian hepatitis B X-interacting protein (HBXIP) enhances the transcriptional activity of NRF2 via a similar mechanism [262].

### GPX4

#### GPX4 in ferroptosis

GPX4 is a glutathione-dependent peroxidase and suppresses both erastin- and RSL3-induced ferroptosis by converting GSH into oxidized glutathione (GSSG) and reducing the cytotoxic lipid peroxides (L-OOH) into the corresponding alcohols (L-OH) [23].

#### GPX4 in breast cancer

In 2007, Udler et al. examined associations between 54 polymorphisms that tag the known common variants (minor allele frequency > 0.05) in 10 genes involved in oxidative damage repair (CAT, SOD1, SOD2, GPX1, GPX4, GSR, TXN, TXN2, TXNRD1, and TXNRD2) and survival in 4470 women with breast cancer. They identified two single nucleotide polymorphisms (SNPs) in GPX4 (rs713041 and rs757229) associated with all-cause mortality, linking the variations in GPX4 to the prognosis of breast cancer [277]. The study conducted by Méplan et al. also suggested a role of polymorphisms of glutathione peroxidase in breast cancer development [278]. In contrast, a previous study showed a negative relationship between polymorphisms in GPX4 and susceptibility to breast cancer [279].

In 2008, Lee et al. demonstrated that GPX4 expression could be upregulated by insulin via the PI3K/Akt pathway in breast cancer cells [280]. The following year, they found that GPX4 could also be upregulated by the E2-induced transient increase in intracellular ROS levels. This E2-mediated GPX4 induction was independent of ERα but associated with the PI3K pathway [281]. In a previous study, E2 was shown to act on mitochondria through anchorage- and integrin-dependent signalling and to generate ROS as signal-transducing messengers to activate the binding of three oxidant-sensitive transcription factors: AP-1, CREB, and nuclear respiratory factor 1 [282]. Thus, insulin and E2 could induce increase ROS, which in turn induced GPX4 expression.

GPXs are differentially expressed in non-cancerous mammary epithelial cells and breast cancer cells. In 2017, Rusolo et al. compared seleno-transcriptome expression between the human non-cancerous mammary epithelial cell line MCF-10A and two human breast cancer cell lines, MCF7 and MDA-MB-231, and identified four downregulated genes, GPX1, GPX4, GPX5 and GPX7, and three upregulated genes, iodothyronine deiodinase 2 (TXDI2), GPX2 and GPX3, in breast cancer cells. They further found 3 HUB nodes interconnected to the differentially regulated selenoproteins, i.e., TP53, ERα and catenin-β1 (CTNNB1), suggesting a need to explore the regulatory mechanism of selenoproteins in breast cancer [283].

### ACSL4

#### ACSL4 in ferroptosis

Of the five isoforms of ACSLs, ACSL3 and ACSL4 activate PUFAs. While ACSL3 prefers OA, ACSL4 favours AA and adrenic acid (AdA). In contrast to other ACSLs, ACSL4 is able to promote ferroptosis by acetylating AA and AdA, which are integrated into the cell membrane by LPCAT3 and induce ferroptosis when oxidized by LOXs and Fenton activity [17, 36].

#### ACSL4 in breast cancer

The expression of ACSL4 in breast cancer tissues has been inconsistent in different studies. In 2016, Chen et al. analysed data from the Oncomine and PrognoScan databases and concluded that ACSL4 was downregulated in breast cancer tissues and the ACSL4 level showed a positive association with the prognosis of patients with breast cancer, in contrast to previous studies [284]. In 2020, Dinarvand et al. compared 55 pairs of fresh samples of BC and adjacent non-cancerous tissue, and they reported that ACSL4 had higher expression in breast cancer tissues than adjacent normal tissue, and its expression level was negatively correlated with Ki-67 and age and positively with the mutant p53 level; however, it was not associated with the expression of ER, PR or HER2 [285].

Maloberti et al. found that ACSL4 could regulate the expression of COX-2 and the production of prostaglandin in MDA-MB-231 cells and that esterification of AA by ACSL4 increased the content of AA in mitochondria, driving specific LOX metabolism of the fatty acid. The interaction between ACSL4, LOXs and COX-2 regulated the proliferating and metastatic potential of breast cancer cells [286]. Interactions between ACSL4, LOX-5 and COX-2 were also confirmed by Orlando et al. [287].

In 2015, Castillo et al. determined the gene expression profile after ACSL4 overexpression in MCF-7 breast cancer cells and identified that ACSL4 was associated with the regulation of embryonic and tissue development, cellular movement and DNA replication and repair [288]. Orlando et al. found that ACSL4 regulated components of the two complexes of the mTOR pathway (mTORC1/2), along with upstream regulators and substrates [289]. Subsequently, they found that ACSL4 promoted cell chemo-resistance in breast cancer cells by regulating expression of transporters involved in drug resistance via the mTOR pathway [290]. Notably, they observed a synergistic effect on cell growth inhibition with the combination of ACSL4 inhibitor rosiglitazone and ERα inhibitor tamoxifen both in ER+ and triple-negative breast cancer cells, indicating an ER independence function of tamoxifen, which has been reported to increase ROS levels and induce premature senescence and cell cycle arrest in breast cancer cells [289, 291].

In vitro, expression of ACSL4 has been reported to be negatively correlated with ER, AR and HER2 in breast cancer cell lines. Overexpression of ACSL4 is able to downregulate ER levels and confer cancer cell growth advantages and resistance to chemotherapy and ER/HER2 targeted therapy [292, 293]. In 2019, Dattilo found that the retinoid-related orphan receptor alpha (RORα), specificity protein 1 (Sp1) and E2F elements were involved in the promoter activity of ACSL4, and oestrogen-related receptor alpha (ERRα) was a transcription factor involved in activation of the human ACSL4 promoter. They also demonstrated that ERα restoration was able to downregulate ACSL4 expression in triple-negative breast cancer cells [294]. In contrast, Belkaid et al. found that 17β-oestradiol treatment upregulated the level of ACSL4 by increasing its half-life, and it promoted an invasive phenotype in an ACSL4-dependent manner in the oestrogen receptor-positive breast cancer cell lines MCF7 and T47D [295].

### P53

#### P53 in ferroptosis

P53 is one of the most studied tumour-suppressor genes, and its inactivation is a common tumourigenic mechanism in multiple cancers including breast cancer. P53 has been shown to manipulate the process of ferroptosis by regulating DPP4, SLC7A11, SAT1, ALOX12, and CDKN1A [25, 26, 296, 297]. TIGAR, GLS2 and SCO2 may also be involved in p53 mediated ferroptosis [298].

P53 has also been reported to regulate cellular ROS and exhibits distinct antioxidant activity in different context. On the one hand, p53 has been reported to increase ROS levels via its target genes such as PUMA, quinone oxidoreductase and a proline oxidoreductase, and the pro-oxidant function has been shown to contribute to p53-induced cell death [299–302]. On the other hand, p53 is also involved in the expression of antioxidant genes such as phosphate-activated mitochondrial glutaminase 2 (GLS2) [303]. Vurusaner et al. claimed that these opposing responses might depend on the cellular levels of p53. In the absence of severe stresses, relatively low levels of p53 are sufficient for the upregulation of several antioxidant genes, maintaining decreased intracellular ROS to protect cells. When the stress exceeds a certain threshold, p53 is upregulated to induce expression of oxidation-inducing genes and increases in cellular ROS [304].

Mutations in p53 impair its function. P53 with mutations at three normally acetylated lysine residues (K117R + K161R + K162R) in the DNA-binding domain leading to a deficiency in acetylation, namely, p53^3KR^, fails to induce cell–cycle arrest, senescence and apoptosis, but remains fully able to regulate metabolic genes and SLC7A11 expression and induce ferroptosis [25]. Nevertheless, the addition of a fourth mutation at lysine K98R in mouse p53 (or K101R for human p53) completely abolishes its ability to regulate metabolic targets, although K98R (or K101R in human) alone modestly affects the function of p53 [305]. These results demonstrated a vital role of acetylation in the complete function of p53, including p53-induced ferroptosis. Liu et al. demonstrated that mutated p53 (R273H or R175H) downregulated expression of system xc^−^ by binding to Nrf2 and thereby retaining NRF2 in the cytoplasm [306]. An African-specific nonsynonymous single-nucleotide polymorphism at codon 47 in TP53 has also been reported to impair its promotion of ferroptosis by increasing levels of coenzyme A (CoA) and GSH [307, 308].

#### p53 in breast cancer

P53 has an antitumour role by inhibiting the cell cycle, regulating apoptosis, promoting DNA repair and inhibiting angiogenesis and metastasis, and it can be inactivated by multiple mechanisms in breast cancer [125].

Previous studies have suggested a role of p53 in ferroptotic death in breast cancer. P53 and ROS can mutually regulate one another and function synergistically. In 2005, Ostrakhovitch et al. demonstrated that activation of p53 played a crucial role in copper and zinc-induced generation of ROS in epithelial breast cancer cells via its target genes such as p53-induced gene 3 product (PIG3) and BAX [309]. Oxidative stress could also upregulate p53 to induce senescence in breast cancer cells [310]. P53 was found to be essential in PARP-mediated necrotic cell death induced by ROS in breast cancer cells [311]. P53 and ROS are both reduced by treatment with vitamin E, an anti-ferroptosis agent [312]. Moreover, wild-type p53 acted as a prooxidant, and mutant p53 was shown to support the survival of breast cancer cells as an antioxidant by regulating expression of thioredoxin (TXN) and HO-1 in a NRF2-dependent manner [313].

### SLC7A11

#### SLC7A11 in ferroptosis

As is well known, SLC7A11 is a subunit of system xc−, which plays a critical role in ferroptosis by importing cystine for the synthesis of GSH [3]. Ferroptosis inducers such as erastin, sulfasalazine and sorafenib are identified as system xc^−^ inhibitors [4, 38, 60].

#### SLC7A11 in breast cancer

RNA sequencing analysis has revealed that SLC7A11 is upregulated in brain metastatic breast cancer tissues, implying a role for SLC7A11 in breast cancer metastasis [314]. In 2017, Ge et al. reported that downregulation of SLC7A11 could confer adriamycin resistance to MCF-7 breast cancer cells by over-expressing P-gp-mediated increased ROS level [315]. In 2018, Bolli et al. showed that inhibition of xCT by virus-like-particle immunotherapy could reduce the metastatic potential of breast cancer stem cells [316]. Vaccines targeting SLC7A11 have been developed and demonstrated to protect mice from mammary cancer metastases [317, 318].

SAS, an anti-inflammatory drug that is used against inflammatory bowel disease and rheumatoid arthritis, was identified as a potent inhibitor of xCT for the first time by Gout et al. in 2001 [38]. Two years later, sulfasalazine was shown to inhibit breast cancer cell proliferation by suppressing cystine uptake [319], and this effect could even be enhanced by inhibiting Insulin-like growth factor I receptor [320]. In 2005, this team found that sulfasalazine could enhance the anticancer effect of doxorubicin by inhibiting xCT and reducing glutathione levels in breast cancer [321]. A similar effect was also demonstrated by Cobler et al. in 2018, who showed that xCT inhibition sensitized breast tumours to γ-radiation via glutathione reduction [322], suggesting a synergic role of xCT inhibition and ROS-generating therapies in killing cancer cells. The anticancer activity of sulfasalazine was also confirmed by Wei et al., who showed that sulfasalazine induced cytotoxicity in MDA-MB-231 breast cancer cells, and the cytotoxicity could be abated by low-dose and enhanced by high-dose vitamin E succinate [323].

In 2011, before the concept of ferroptosis was raised, SLC7A11 was reported to be downregulated by microRNA-26b, inducing apoptosis in MCF7 and HCC1937 but not MDA-MB-231 breast cancer cells [324]. Notably, the “apoptosis” in this study was assessed by the TUNEL assay, but not markers such as the caspase family or Bcl2.

In 2013, a functional metabolic portrait of 46 independently derived breast cell lines was illustrated by Timmerman et al., who identified a subset of triple-negative samples that were glutamine auxotrophs. xCT was expressed on one-third of triple-negative tumours in vivo and could be inhibited by sulfasalazine, suppressing the growth of breast cancer cells [99].

In 2015, Habib et al. found that transcription of xCT could be upregulated by NRF2, while KEAP1 contributed to the ubiquitination of NRF2. In response to oxidative stress, breast cancer cells could upregulate xCT via the KEAP1/NRF2 pathway to antagonize ROS in cells [268]. In contrast, knockdown of SLC7A11 led to increased ROS levels in breast cells [325]. xCT could also be regulated by MUC1-C and CD44 variant (CD44v), which interacts with xCT and thereby controls GSH levels and protects triple-negative breast cancer cells against treatment with erastin [101, 102].

Expression of xCT confers cells relative resistance to ferroptosis, but under glucose-deficient/glutamine-replete conditions, downregulation of xCT improves cell viability by enhancing the ability of cells to use intracellular glutamate to maintain mitochondria respiratory chain activity [325].

## Conclusion and future perspectives

As a newly raised type of cell death, ferroptosis has not been extensively studied in breast cancer. In this review, we compared ferroptosis with other common cell death patterns and reviewed the core regulators of ferroptosis from a historical perspective with a focus on the novel death pattern of ferroptosis. By reviewing the ferroptosis-like phenomenon in previous studies and comparing it with other types of cell death, we can better understand both ferroptosis and its regulators. Mesenchymal-high TNBC cells and therapy-resistant cells have been shown to be more susceptible to ferroptosis due to their metabolic features and cellular signalling pathways, making ferroptosis a promising candidate to overcome this refractory issue.

To date, ferroptotic therapeutic strategies such as CSO-SS-Cy7-Hex/SPION/Srfn, erastin@FA-exo, HMCMs, DFTA and NFER have been developed and applied to research in breast cancer treatment. It is reasonable to believe that ferroptotic therapeutics may improve the prognosis of patients with breast cancer in the future.

However, compared with the problems solved, additional mysteries are waiting to be discovered. What is the biological marker of ferroptosis similar to caspases in apoptosis? Which step of ferroptosis makes the progress irreversible? What is the exact mechanism by which lipid ROS induces cell lysis? How can target cancer cells be more accurately and efficiently targeted by inducing ferroptosis according to the different types of metabolism between cancer and normal cells? How can ferroptotic agents be delivered to cancer cells through the microenvironment? How can resistance to ferroptosis be overcome in ER+ breast cancer? Can TNBC be edited to become more sensitive to ferroptosis? Is there any more crosstalk between such diverse kinds of cell death? How can different types of cell death be combined to enhance lethality to cancer cells? Are there additional ferroptosis inducers and regulatory genes that remain to be discovered? Can more FDA-proved agents induce ferroptosis and be applied clinically? How can side effects caused by ferroptotic agents be reduced in the clinic? Many problems must be solved before ferroptosis can be applied in the clinic.

## Data Availability

Not applicable.

## Abbreviations

ROS: Reactive oxygen species
GPX4: Glutathione peroxidase-4
PUFA: Polyunsaturated fatty acid
xCT: SLC7A11
ACSL4: Acyl-CoA Synthetase Long Chain Family Member
IRI: Ischaemia reperfusion injury
SAS: Sulfasalazine
TFRC: Transferrin receptor
DMT1: Divalent metal transporter 1
ARF: Cyclin Dependent Kinase Inhibitor 2A
ATG5/7: Autophagy-related 5/7
HO-1: Heme Oxygenase 1
HSPB1: Heat Shock Protein Family B (Small) Member 1
ER: Oestrogen receptor
FSP1: Ferroptosis suppressor protein 1
MTDH: Metadherin
GSH: Glutathione
GSSG: Glutathione disulfide
GR: Glutathione reductase
GSS: Glutathione Synthetase
GCL: Glutamate-cysteine ligase
Nec-1: Necrostatin-1
DFO: Deferoxamine
MVBs: Multivesicular bodies
AA: Arachidonic acid
AdA: Adrenic acid
LPCAT3: Lysophosphatidylcholine acyltransferase 3
LA: Linoleic acid
CoA: Coenzyme A
ECM: Extracellular matrix
TAG: Triacylglyceride
ATGL: Adipose triglyceride lipase
RIPK1: Receptor Interacting Serine/Threonine Kinase 1
RIPK3: Receptor Interacting Serine/Threonine Kinase 3; eukaryotic translation initiation factor 2 alpha kinase 4
eIF2α: Eukaryotic translation initiation factor 2 subunit alpha
ATF4: Activating transcription factor 4
PUFA-PL: Polyunsaturated fatty acid containing phospholipid
SCD1: Stearoyl-CoA desaturase
MUFA: Monounsaturated fatty acid
FBP1: Fructose-1,6-biphosphatase
TRIP-Br1: Transcriptional regulator interacting with the PHD-bromodomain 1
PI3K: Phosphatidylinositol 3-kinase
GCN2, DHA: Docosahexaenoic acid
XIAP: X-linked inhibitor of apoptosis protein
BAX: BCL2-associated X protein
APAF-1: Apoptotic peptidase activating factor 1
PUMA: P53-upregulated modulator of apoptosis
p53AIP1: P53-regulated apoptosis-inducing protein 1
PIDD: P53-induced death domain protein 1
NOXA: Phorbol-12-Myristate-13-Acetate-Induced Protein 1
DT: Dihydroisotanshinone I
PEITC: Phenethyl isothiocyanate
RIP: Receptor-interacting protein
MLKL: Mixed lineage kinase domain-like pseudokinase
CDDO: 2-amino-5-chloro-N, 3-dimethylbenzamide
HSP90: Heat shock protein 90
NCOA4: Nuclear receptor coactivator 4
CMA: Chaperone-mediated autophagy
ATP: Adenosine triphosphate
LPS: Lipopolysaccharide
PAMPs: Pathogen-associated molecular patterns
DAMPs: Damage-associated molecular patterns
GSDMD: Gasdermin D
DHA: Docosahexaenoic acid
15d-PGJ2: 15-Deoxy-Δ12,14-prostaglandin J2
PPAR: Peroxisome proliferator-activated receptor
HO-1: Haem oxygenase-1
RNR: Ribonucleotide reductase
Hb: Haemoglobin
SFAs: Saturated fatty acids
OR: Odd ratio
CI: Confidence interval
EPA: Eicosapentaenoic acid
OA: Oleic acid
GLA: γ-linoleic acid
COX: Cyclooxygenases
LOXs: Lipoxygenases
NAC: N-acetyl-cysteine
GCH1: GTP Cyclohydrolase 1
BH_4_: Tetrahydrobiopterin; (1S, 3R)RSL3, (1S, 3R)-RAS synthetic lethal 3
src: Proto-Oncogene Tyrosine-Protein Kinase Src
STAT3: Signal transducer and activator of transcription 3
ACLS4: Acyl-CoA Synthetase Long Chain Family Member 4
PVRL4: Also known as Nectin-4, Nectin Cell Adhesion Molecule 4
ERα: Oestrogen receptor α
E2: 17β-estradiol
FPN1: Ferroportin-1
CYP: Cytochrome P450 monooxygenases
ETC: Electron transport chain
NOX: Nicotinamide adenine dinucleotide phosphate hydride (NADPH) oxidases
MVA: Mevalonate
HMGCR: HMG-CoA reductase
HMG-CoA: 3-hydroxy-3-methyl-glutaryl-coenzyme A
IPP: Isopentenyl pyrophosphate
Se: Selenium
CL: Cardiolipin
RXR: Retinoid X receptor
OSGIN1: Oxidative stress-induced growth inhibitor 1
DMBA: 7,12-dimethylbenz (aanthracene
Rp: Rapamycin
GPER1: G protein coupled oestrogen receptor 1
N-SMYase: Neutral sphingomyelinase
TRPC: Transient receptor potential canonical
NRF2: Nuclear factor E2-related factor 2
KEAP1: Kelch-like ECH-associated protein 1
NQO1: Quinone oxidoreductase 1
FTH1: Ferritin heavy chain 1
ARE: Antioxidant responsive element
GST: Glutathione S-transferase
PEITC: Phenethyl isothiocyanate
DPP: Dipeptidyl-peptidase
HBXIP: Hepatitis B X-interacting protein
SNPs: Single nucleotide polymorphisms
RORα: Retinoid-related orphan receptor alpha
ERRα: Oestrogen-related receptor alpha
GLS2: Glutaminase 2
PIG3: P53-induced gene 3 product
TXN: Thioredoxin
